# A workflow for streamlined acquisition and correlation of serial regions of interest in array tomography

**DOI:** 10.1186/s12915-021-01072-7

**Published:** 2021-07-30

**Authors:** Sergio Gabarre, Frank Vernaillen, Pieter Baatsen, Katlijn Vints, Christopher Cawthorne, Steven Boeynaems, Emiel Michiels, Dorien Vandael, Natalia V. Gounko, Sebastian Munck

**Affiliations:** 1 VIB-KU Leuven Center for Brain & Disease Research, Electron Microscopy Platform & VIB BioImaging Core, O&N5 Herestraat 49 box 602, 3000 Leuven, Belgium; 2 KU Leuven Department of Neurosciences, Leuven Brain Institute, O&N5 Herestraat 49 box 602, 3000 Leuven, Belgium; 3 VIB-KU Leuven Center for Brain & Disease Research, Light Microscopy Expertise Unit & VIB BioImaging Core, O&N5 Herestraat 49 box 602, 3000 Leuven, Belgium; 4 VIB BioInformatics Core, Technologiepark 75, 9052 Ghent, Belgium; 5 MoSAIC-Molecular Small Animal Imaging Centre, KU Leuven, Leuven, Belgium; 6 Department of Genetics, Stanford University School of Medicine, Stanford, California, 94305 USA; 7 VIB Center for Brain and Disease Research, 3000 Leuven, Belgium; 8 Switch Laboratory, Department of Cellular and Molecular Medicine, KU Leuven, 3000 Leuven, Belgium

**Keywords:** Array tomography, Integrated light and electron microscope, 3D EM, CLEM, Correlation, Open source, Golgi staining, workflow, Smart microscopy, Auto-navigation

## Abstract

**Background:**

Array tomography (AT) is a high-resolution imaging method to resolve fine details at the organelle level and has the advantage that it can provide 3D volumes to show the tissue context. AT can be carried out in a correlative way, combing light and electron microscopy (LM, EM) techniques. However, the correlation between modalities can be a challenge and delineating specific regions of interest in consecutive sections can be time-consuming. Integrated light and electron microscopes (iLEMs) offer the possibility to provide well-correlated images and may pose an ideal solution for correlative AT. Here, we report a workflow to automate navigation between regions of interest.

**Results:**

We use a targeted approach that allows imaging specific tissue features, like organelles, cell processes, and nuclei at different scales to enable fast, directly correlated in situ AT using an integrated light and electron microscope (iLEM-AT). Our workflow is based on the detection of section boundaries on an initial transmitted light acquisition that serves as a reference space to compensate for changes in shape between sections, and we apply a stepwise refinement of localizations as the magnification increases from LM to EM. With minimal user interaction, this enables autonomous and speedy acquisition of regions containing cells and cellular organelles of interest correlated across different magnifications for LM and EM modalities, providing a more efficient way to obtain 3D images. We provide a proof of concept of our approach and the developed software tools using both Golgi neuronal impregnation staining and fluorescently labeled protein condensates in cells.

**Conclusions:**

Our method facilitates tracing and reconstructing cellular structures over multiple sections, is targeted at high resolution ILEMs, and can be integrated into existing devices, both commercial and custom-built systems.

**Supplementary Information:**

The online version contains supplementary material available at 10.1186/s12915-021-01072-7.

## Background

Advances in 3D electron microscopy (EM) imaging [1] and correlative and multimodal imaging have revolutionized life science imaging [2]. 3D EM imaging has been achieved in a number of ways [3–7], including so-called array tomography (AT) where serial sections are imaged with an SEM [8, 9].

Correlative light and electron microscopy (CLEM) combines the specificity and flexibility of light microscopy (LM) with the ultrastructural context and comprehensive information available via EM [10]. However, a key challenge is the overlaying of LM and EM outputs to produce the final correlated image, as the resolution gap between LM and EM and different distortions in the two techniques prevent straightforward automation [11].

Integrated light and electron microscopes, so-called iLEMs, have been designed to overcome the problem of alignment between the two modalities for imaging in both LM and EM [12–16]. Different realizations of iLEMs have been developed, including transmitted and scanning electron microscopes [12, 13, 16–19] and setups dedicated towards 3D imaging [20]. For AT, the use of a high-resolution iLEM that uses diffraction-limited oil immersion lenses (with a resolution of about 200 nm in the *x,y* range and that can even be used for super-resolution [21]) combined with a high-resolution SEM seems the most promising for bridging the gap between LM and EM imaging smoothly.

iLEM imaging datasets can be pre-aligned using, for example, cathodoluminescence on the nanometer scale [22]. Correlation is done in the plane of each section, limiting the correlation problem to 2D and reducing any inaccuracy in Z-direction to the thickness of the section and even beyond the limit of resolution of the LM in Z-direction. Given that on iLEMs, typical ultrathin sections are imaged, these devices are the ideal candidate for correlative AT [17]. However, so far, correlative AT using an iLEM system has not been widely adopted [17, 23].

Generally, software solutions for correlating images using AT [24–26] are not designed for iLEMs, and therefore, integrating acquisition workflows has not been explored. Additionally, the fluorescence signal of a reporter is required as the basis for navigation to specific areas of interest [27].

We reasoned that for imaging tissue and cells in AT there is an unmet need to rapidly and reliably identify similar structures in regions of interest (ROIs) in consecutive sections independent of fluorescence to facilitate 3D imaging of specific structures. As the manual extraction of structures across multiple sections in the tissue context and their re-identification in subsequent sections is time-consuming, we developed a semi-automated workflow for AT based on a high-resolution iLEM.

Our targeted workflow can efficiently image selected structures like organelles and cellular processes, in tissue without requiring fiducials, and is independent of the nature of the signals, to create 3D reconstructions from regions of interest (ROIs). The navigation starts with a brightfield microscopy “overview” image, for which we integrated a transmitted light source in our iLEM. The overview with all the ribbons is used to consequently identify and normalize the individual sections and calculate the coordinates of the ROIs across the sections. Consequently, navigation parameters are refined and localization accuracy is optimized by automatic and fast navigation along consecutive sections in several rounds of stepwise increasing magnification at LM- and EM-level.

Although our workflow is implemented on a commercial iLEM, it can be reused generically, and the associated software, called Tomo (Japanese for “friend”), is open source. Compared to current commercial solutions, our workflow is faster, efficient, and more accurate due to the overlay of smaller fields of view.

As a proof of concept, we demonstrate our approach on Golgi impregnated tissue samples and correlate transmitted LM and EM for guided acquisition AT and on cells expressing fluorescent condensates.

## Results

Here, we present a novel approach that allows creating 3D reconstructions from regions of interest (ROIs) of cut tissue sections utilizing an iLEM by semi-automated navigation to these ROI across ribbons of the cut sections. Our approach seamlessly integrates into standard preparation procedures for iLEM imaging.

**Fig. 1.**
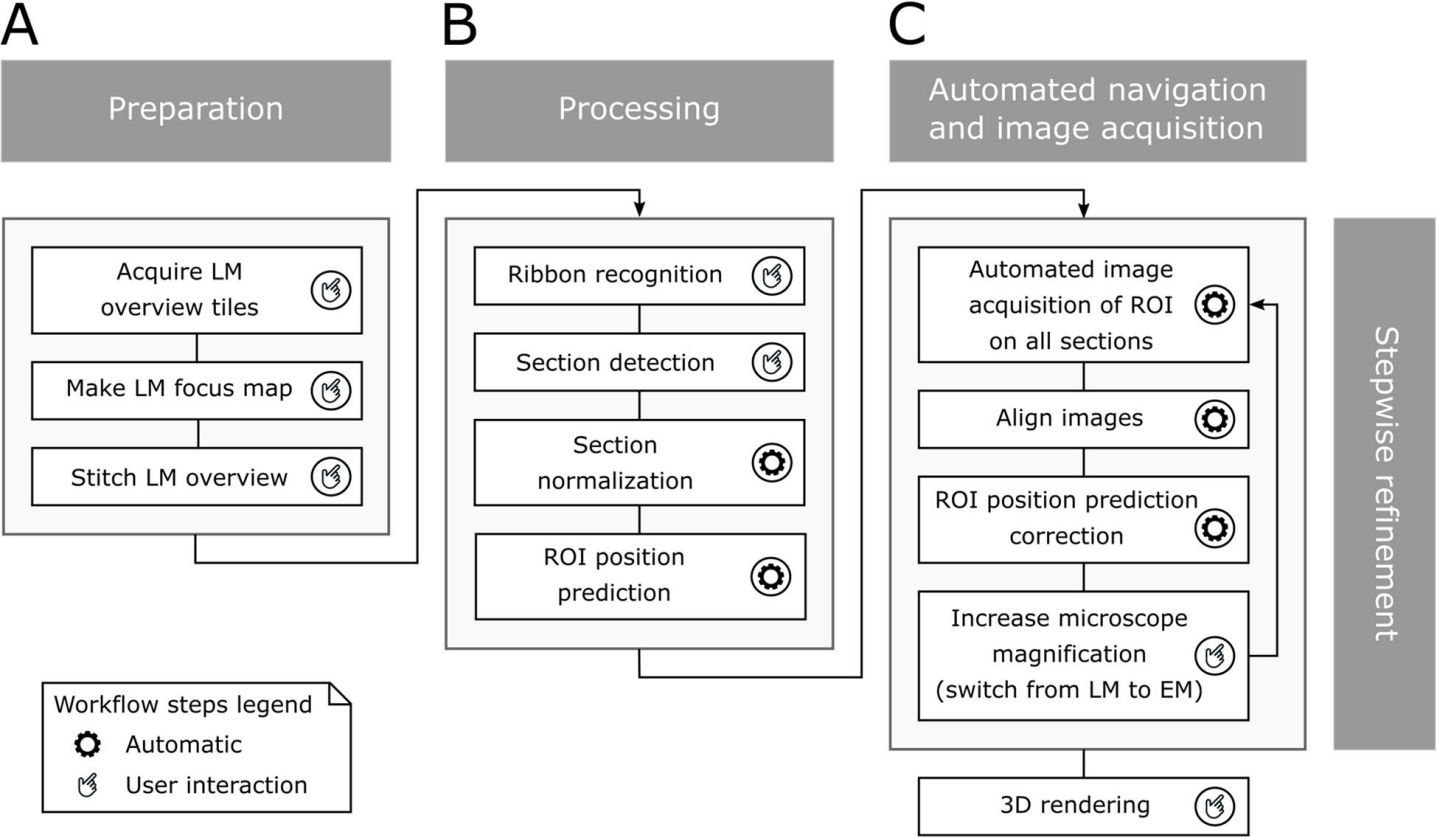
Detailed imaging workflow. **a** Preparation. To acquire the (correlative) LM/EM image datasets on the iLEM, first a tiled overview image is created using a 20x lens and transmitted light, then the stage coordinates are aligned to overview image pixel coordinates and a focus map is built. **b** Processing. Using image analysis tools, the ribbons are recognized and the sections detected. Additionally, a normalization procedure corrects for shape changes of the sections due to cutting and other factors. Using these processing steps, the coordinates of the ROI are predicted across the ribbons, with ROIs being first defined on the overview image, by selection in one section. **c** Automated navigation and acquisition. Using the designated coordinates image stacks are acquired. After acquisition, the stack is registered, and the correction used can be applied to update the coordinates of the ROI. This stepwise refinement can be repeated with each switch of magnification and modality (100x LM, 4kx, 12kx, etc.)

**Fig. 2.**
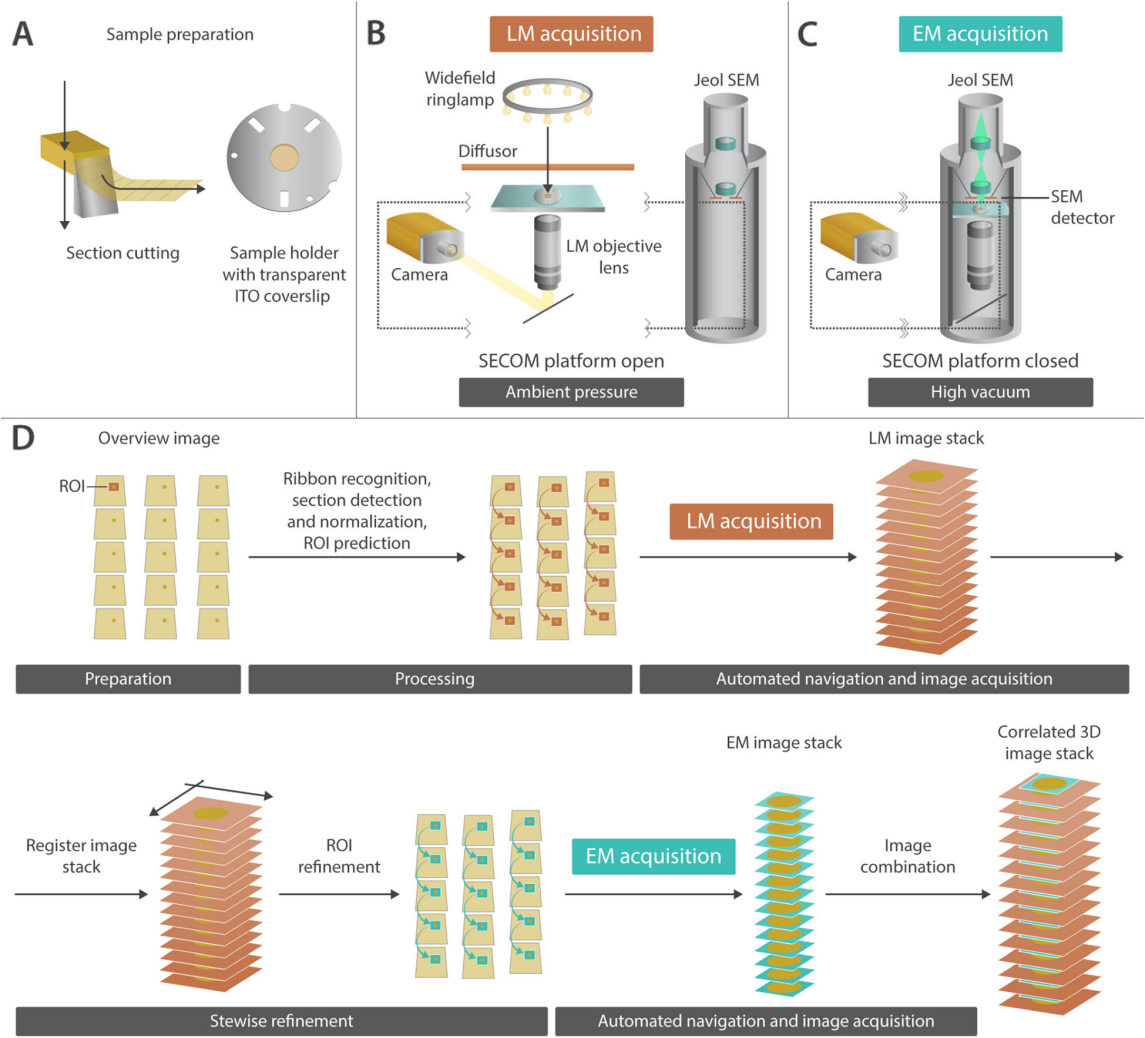
Experimental overview of our targeted AT iLEM approach. **a** sample preparation. The tissue block is sliced on an ultramicrotome and mounted on the sample holder using an ITO coverslip. **b** LM acquisition. A transmitted light source was added to generate an overview image consisting of a mosaic of transmitted light images using a 20x optical lens. Additional 100x imaging is applied. LM imaging is carried out at ambient pressure. **c** EM acquisition. A JEOL SJF7200 SEM retrofitted with the commercial SECOM platform was used. **d** introduces key steps of the workflow. The workflow can be divided into (i) preparation (overview image and focus map creation), (ii) processing (ribbon detection, section recognition, normalization of section geometries for the prediction of the position of ROIs in consecutive sections), (iii) automated navigation and image acquisition (with increasing magnifications), and (iv) stepwise refinement (using a feedback mechanism for the navigation based on the image alignment at each magnification step). For more information on the workflow, see Fig. 1

Figure 1 illustrates the complete acquisition workflow, while Fig. 2 provides the overview and connects our approach with a general experimental set up.

The workflow is divided into four building blocks: (i) preparation (overview image and focus map creation, (ii) processing (ribbon detection, section recognition, normalization of section geometries for the prediction of the position of ROIs in consecutive sections), (iii) automated navigation and image acquisition (with increasing magnifications at 20x LM, 100x LM, and at 4kx EM and 10-12kx EM), and (iv) stepwise refinement (using the information from aligning the images of the ROIs at each magnification step and for refining the navigation for the next magnification).

The workflow is designed to accommodate imperfections from the sample preparation process (e.g., variations introduced by the cutting, changes in section shape) and overcomes the difficulties of identifying the correct ROI position across sections (for documentation and a user guide with step-by-step explanations of our software solution, please see Additional file 1: Text S1 and Additional file 2 Text S2, Technical documentation and User manual of Tomo, respectively). In the following, we elaborate on the individual steps in more detail to describe our proof-of-concept.

### Workflow

#### I. Preparation

##### Overview image

**Fig. 3.**
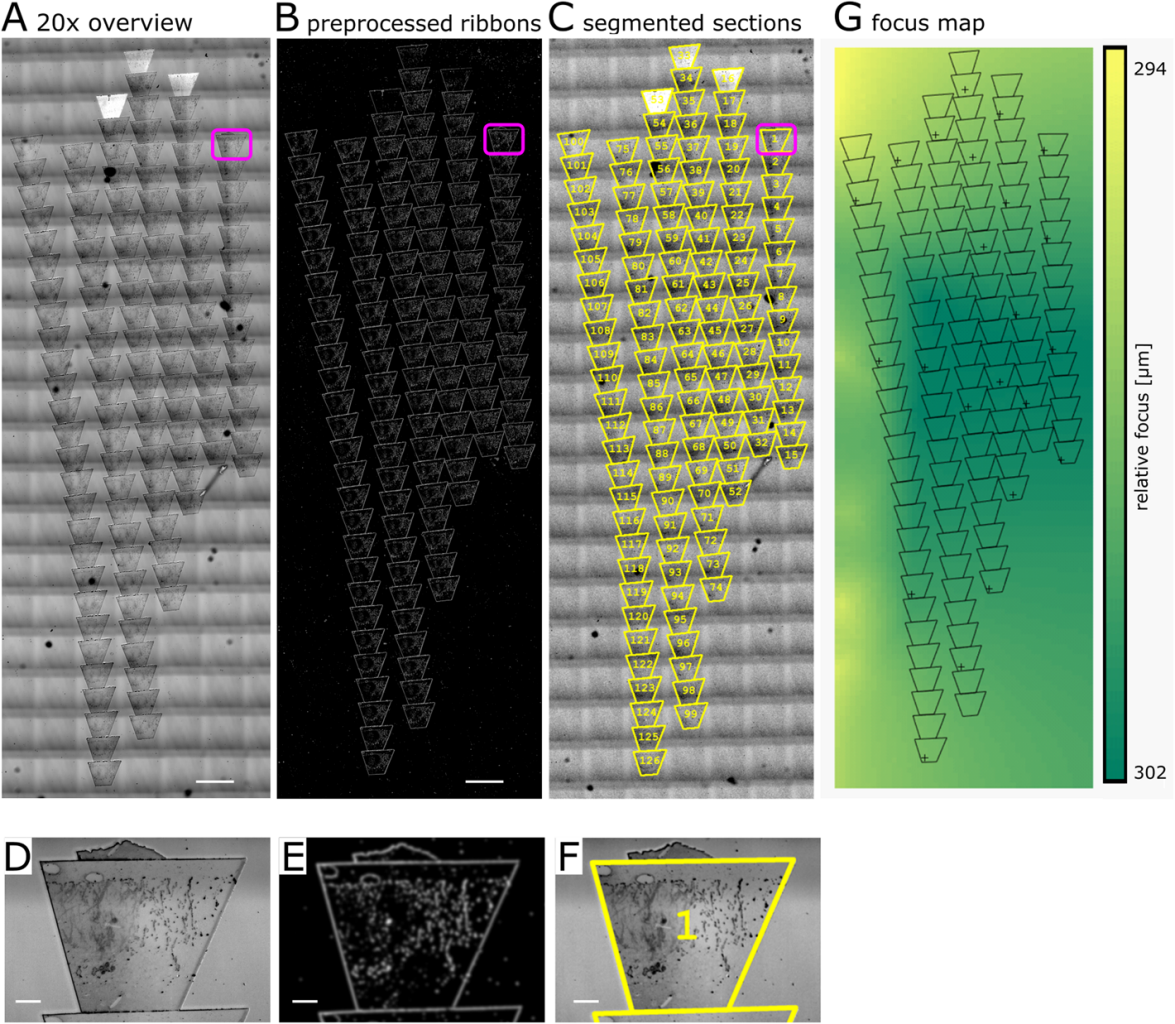
Overview image, processing for ribbon detection, section recognition, and focus map acquisition. **a** 20x LM overview image, **b** Preprocessed ribbons in LM (see also supplementary Figure 1 for individual steps). **c** Segmented sections; overlaid onto the overview image. **d**–**f** Close-up of the first section as indicated by the magenta rectangle. **g** Example focus map showing manual focus positions (+). These can be updated during the complete process if needed. The color code is indicating the interpolated focus z values. Scale bar in **a**–**c** = 500 μm. Scale bar in **d**–**f** = 50 μm

First, we generated an overview navigation image, i.e., an overview image that is linked to the coordinates of the stage. As our iLEM does not provide absolute stage coordinates, we introduced an additional step to “calibrate” the stage position. The relative movements of the translation stage of the microscope were converted into absolute coordinates in our software by selecting a section corner near the center of the field of view (FOV) and setting the coordinates to 0,0. Consequently, the coordinates are connected to the overview image. This means that we do not need to re-image the sections at any later step but can use the coordinates directly or refine them in the process of imaging at higher magnification. To generate this overview image, the whole sample with the distribution of all sections in the form of ribbons that are deposited onto the conductive ITO coverslip needed to be acquired with transmitted light. Since our iLEM is lacking a transmitted light source, we used an external ring lamp and a diffuser to illuminate the sample uniformly, allowing us to acquire these brightfield images (Fig. 2). The individual images for the overview were acquired with overlapping borders using a 20x dry lens. In Fig. 3, the outlines of the sections can be well distinguished.

##### Focus map creation

In order to guarantee optimal focus during the imaging process, a focus map was created. For this purpose, reference points in every ribbon are selected by the user. The corresponding focus values are used by the application to calculate and interpolate focus planes for the optical microscope to acquire the images through consecutive sections and, as such, reduce the need for user input (Fig. 3). To create a proper focus map, several interpolation methods were tested. We found that “natural neighbor interpolation” [28] (see Additional file 1:Text S1, Technical Documentation) yielded acceptable results. It is of note that this focus map will be used at all levels of the LM acquisition. For EM imaging, the autofocus routine of the microscope itself is employed in addition.

##### Stitching

Stitching of the 20x overview images with 20% overlap was carried out using Microscopy Image Stitching Tool (MIST) or Grid/Collection stitching in Fiji/ImageJ [29–31].

#### II. Processing

##### Ribbon detection

Next, the (stitched) overview navigation image is used as the starting point to detect ribbon boundaries and consequently recognize the sections. For that purpose, several straightforward preprocessing steps like simple thresholding, size filtering, and sample smoothing to account for occasional variations in the thickness of the sections can be applied to mark the ribbons (for an overview of these preprocessing steps applied to the overview navigation image in our proof of concept, see Additional file 3: Figure S1). These processing steps are bundled into a “preprocess” tool within Tomo to support the rough detection of the ribbons (Fig. 3 and Additional file 1: Text S1, Technical Documentation).

##### Section recognition

Based on the detected ribbons, we applied an active contour method [32] that uses an optimization algorithm where an initial quadrilateral shape of the section is modified automatically to match the boundaries of the actual section based on the preprocessed overview navigation image. The active contour method’s adaptiveness then allows approximating the individual section shape (Fig. 3, Additional file 3: Figure S1, and for more details, Additional file 1: Text S1, Technical Documentation).

To confirm the robustness of the section detection, we tested different conditions simulating various forms of sample degradation, i.e., noise and lack of corners (Additional file 4: Figure S2). We found that while varying the signal-to-noise ratio by adding Gaussian noise with standard deviations of 10, 40, and 70 to the 8-bit gray levels of the original overview navigation image, we could still reliably detect the contours and frame them within an accuracy of 60 micrometers. In addition, in the presence of salt and pepper noise, the sections could still be detected. To test how important the corners of the sections are for our section recognition approach, we also tested images where 1-4 corners/1-4 section sides had been deleted. We found that this still resulted in sections being detected correctly.

##### Normalization of section geometries for the prediction of the position of ROIs in consecutive sections

**Fig. 4.**
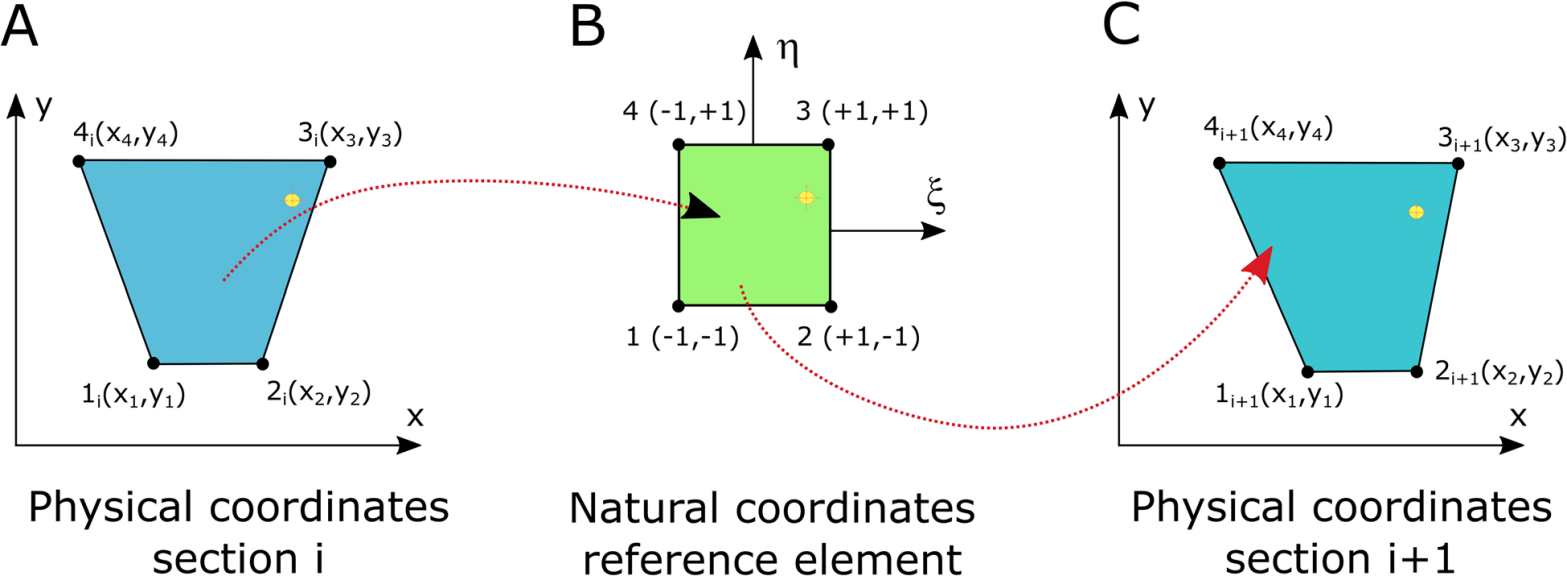
Section normalization and loci propagation of the ROIs. A transfinite transformation is used to predict the position of an ROI (yellow dot) through consecutive sections at the level of the overview image. **a** Position of the ROI at a certain section *i*. **b** Transformation of the ROI position and section corners in a standard shape. **c** Prediction of the ROI in section i+1 is calculated from the corner positions at i+1 and the ROI position in the standard section

Next, to predict the position of an ROI in consecutive segmented sections and compensate for potential errors arising from any shape deformation between them, we used an algorithm based on the geometrical transfinite transformation used in Finite Element Formulations normalization [33]. Based on a quadrilateral base element, every section is transformed into a square expressed in natural coordinates with a linear transformation between Cartesian coordinates (x,y) and natural coordinates (ξ,η). This mapping enabled the bidirectional transformation of each section into a reference section. Hence, it allows the prediction of the position of the ROIs, independent of the precise shape of the section, by using the relative position in one section and transferring it to the next section through a geometric transformation (Fig. 4; for further description of the equations used, see Additional file 1: Text S1, Technical Documentation).

#### III. Automated navigation and image acquisition

**Fig. 5.**
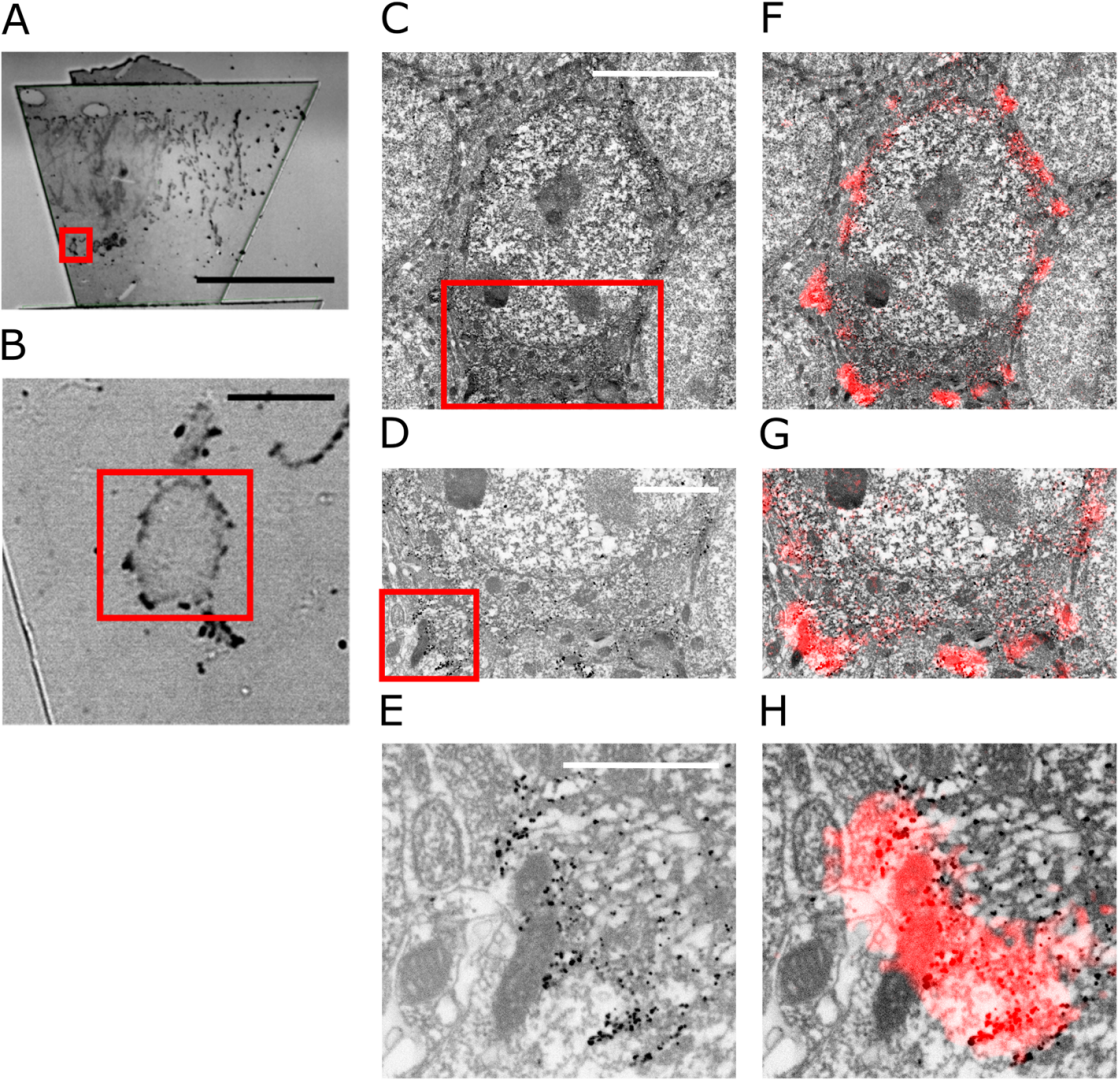
Overview of the images acquired at one location with different magnifications and their combination/correlation. **a** 20x transmitted light image. The section is visible, and the red frame is indicating a region of interest containing one Golgi impregnated cell. **b** 100x LM transmitted light image of a Golgi impregnated neuron of the red area in **a**. **c** 4kx EM image of the region indicated by the red frame in **b**. **d** 12kx EM image of the red square in **c**. **e** 30kx image. Zoom to the region of the red square in **d**. **f**, **g**, **h** Overlay of **b** with **c**, **d**, and **e**, respectively (using ec-CLEM [11]). The LM information is displayed as a false-color image with red indicating the Golgi impregnation deposit in **a** and **b** to overlay and correlate it with the EM information. The figure illustrates how the stepwise refinement, by using the image registration information as feedback, at each magnification allows imaging the same location at different magnifications. This allows acquiring a pyramid of images at different magnifications that scales between more context and more detail in opposing directions. The images of the different magnifications and modalities can then be combined and correlated. Pixel sizes: 20x (2.96 [pixel/μm]) and 100x (15.38 [pixel/μm]) for the LM, 4kx (43.03 [pixel/μm]), 12kx (129.08 [pixel/μm]), and 30kx (322.69 [pixel/μm] for the EM. Values refer to the *x,y*-plane. Scale bars: **a** 20x = 200 μm; **b** 100x = 10 μm; **c** 4kx = 5 μm; **d** 12kx = 2 μm; **e** 30kx = 1 μm

ROIs are first defined on the overview image by selection in one section. Based on the positions of the ROIs, a list of coordinates is generated for the acquisition of an image series using the normalization mentioned above. Consequently, this series of images originating from the same ROI in consecutive sections comprises a 3D image stack. This list of coordinates is used to drive the stage of the microscope to the predicted loci in the individual sections and acquire the images automatically in the right order in the consecutive sections across the ribbons. The idea is to automatically acquire the image stack by moving to the right coordinates and do this for all magnification steps (20x LM, 100x LM, 4kx EM, 10-12kx EM, and eventually 30kx EM; Fig. 5). In our setup, the LM modalities are acquired at ambient pressure, as either transmitted light is used or GFP is quenched by high vacuum [21]. The series of images, stacked together, contains the 3D information of the ROIs. The user can select the starting section and define how many sections to include in the imaging in Tomo (see Additional file 2: Text S2, User Manual). The navigation file with the coordinates can be reused if the acquisition process is interrupted or more ROIs are imaged. The automated acquisition is repeated at all subsequent magnification levels.

#### IV. Stepwise refinement

We reasoned that with increasing magnification, the absolute errors in alignment of the image stack obtained from the consecutive sections would be magnified and that this will affect the performance. Consequently, we thought the smallest improvements could have a marked effect on the final acquisition of the EM images. Therefore, the cornerstone of our automatic image acquisition pipeline is a stepwise refinement of the alignment with each magnification step, ranging from the low magnification navigation image (20x LM lens) to the final high magnification image of 30kx acquired in the electron microscope (see Fig. 5). As a result, this leads to improved accuracy and to pyramidal volumes of images of the ROI.

To this end, at each step, the sequence of images from the ROIs of the different sections is aligned (registered) using standard tools like SIFT [34] or StackReg [35] plugins in Fiji/ImageJ [28]. After checking the alignment/registration of the images in the stack, the geometrical transformations that were needed for image registration are then applied to the ROI coordinates to correct the ROI position and to correct the navigation to the ROIs at the next magnification. This feedback loop of refinement based on the image registration, which is integrated into Tomo, can be applied automatically to update the coordinates in a navigation file for the next round of automatic acquisition.

In our case, after the acquisition and alignment of the 20x and 100x LM image stacks, the EM chamber is evacuated for EM imaging at 4kx in EM (Fig. 5). While Tomo currently tracks only a single ROI across multiple sections, the ROI position predictions can be made completely independent of one another, and hence, our workflow perfectly allows for the acquisition of several ROIs in parallel so that the first LM steps of several regions can be acquired before switching to EM mode. Like this, the timing of the acquisition can be optimized with respect to the waiting times for the evacuation of the chamber, and multiple regions can be multiplexed.

### Experimental testing

#### Proof-of-concept

**Fig. 6.**
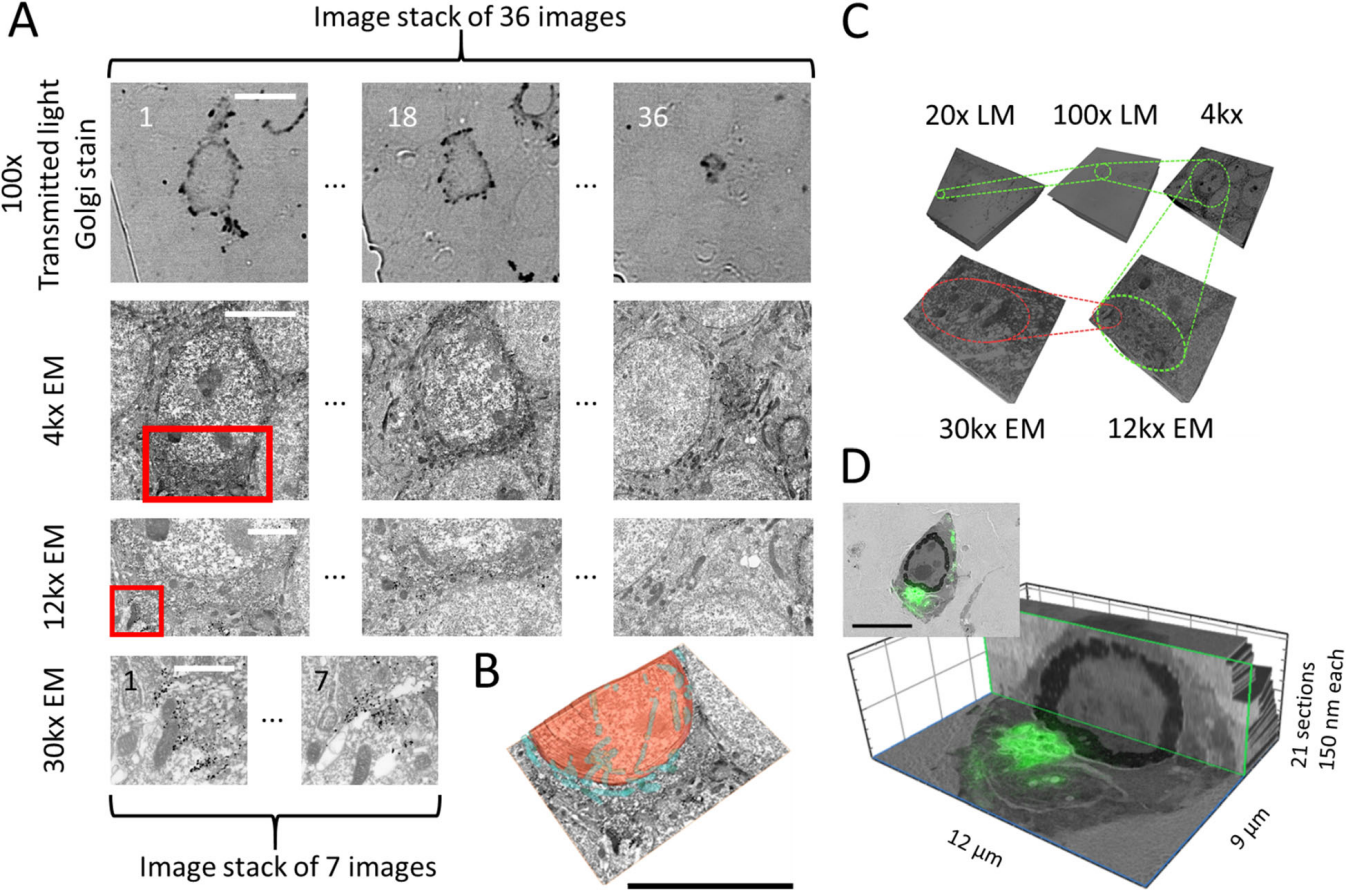
An example of the self-guided navigation of ROIs with automated LM/EM acquisition. **a** Golgi impregnated tissue sections. Example of a dataset tracked over 36 sections 3 example images imaged with 100x, 4kx, 12kx, and 2 example images of the 7 images tracked at 30kx are shown. **b** Manual segmentation of the cell body, nucleus, and mitochondria at 12kx using the EM information at 12kx only. **c** Overview and spatial relation of the self-guided navigation of the 3D stacks shown in **a**. **d** Example of fluorescent dataset acquired with our approach. 100x LM fluorescent images are overlaid on a 4kx image (insert) and the 10kx EM image stack rendered in 3D. The phase separation of FLOE1-GFP fusion protein is shown in U2OS cells. Pixel sizes in **a**: 100x (15.38 [pixel/μm]) for the LM, 4kx (43.03 [pixel/μm]), 12kx (129.08 [pixel/μm]), and 30kx (322.69 [pixel/μm] for the EM. Pixel sizes of EM in **d**: 4kx overview (43.03 [pixel/μm]) and 10 k main panel (107.58 [pixel/ μm]) LM same as in 100x in **a**. Values refer to the *x,y*-plane. Scale bars in **a** 100x = 10 μm; 4kx = 5 μm; 12kx = 2 μm; 30kx = 1 μm. **b**, **d** = 10 μm

To test our approach, we used our recently improved Golgi staining procedure [36]. Golgi staining labels individual cells in (mouse) brain tissue (Figs. 5 and 6), is compatible with LM and EM imaging, and can be visualized straightforwardly at all magnifications ranging from the 20x transmitted light overviews to the high magnification EM imaging; such Golgi-stained tissue therefore provides an ideal opportunity to test our method. In addition, as this staining does not bleach easily it allowed us to optimize individual steps without the need for new samples. In addition, as EM compatible fluorescent labeling in tissue still is non-trivial we wanted to emphasize the possibility to use histochemistry-based approaches instead.

The sample was sectioned, and ribbons of 126 consecutive 150 nm thick sections were collected on an ITO coverslip, in 6 ribbons of each 15 to 27 sections. Then, we acquired an overview navigation image of 7 × 20 image tiles with an overlap of 20%. Our active contour approach detected 126 of the 126 sections. We applied our stepwise refinement approach with 20x, 100x optical, and 4kx, 12kx, and 30kx electron imaging magnification. Using that method, we followed ROIs over 36 sections with a magnification of 12kx, and we were able to trace cellular compartments like the nuclear envelope. To show the potential of the gained information, we also segmented the EM volume (manually) at 12kx (36 sections) and reconstructed it in 3D (Fig. 6b and Additional file 5: Movie S1). For the segmentation, we used the EM information only. In addition, we show two examples of 7 sections highlighting the complete width of the depicted organelles at 30kx magnification (Fig. 6). Additional file 6: Movie S2 and Fig. 6c highlight the combination of the image stacks and their spatial relation as 3D connected pyramidal volumes at a different resolution.

To estimate the amount of correction needed for the navigation to the individual ROIs across the magnifications, we used the 12kx EM images as ground truth. Based on this, we plotted the progressive navigational improvements across the stepwise refinement approach. Additional file 7: Figure S3 shows an example of the refinement of the auto-navigation using one specific ROI along the sections. We can show that our refinement corrects up to 20 μm in this example for the 30th section. The error was calculated as $\sqrt{x^2+{y}^2}$, with x and y being 17.01 μm and 9.47 μm for the 20x lens. We compared the movement predicted for navigation at every step: the semi-automatic detection of the sections at the overview image, the automatic acquisition and the stack alignment of the LM images with the 100x lens, and finally, after the first EM stack at 4kx. The absolute position errors were reduced at every step of the pyramid.

#### Suboptimal samples

Next, we tested our workflow for robustness using two non-ideal samples (see Additional file 8: Figure S4). The first sample consisted of six ribbons with a total of 75 sections of good quality at the boundaries but with ribbons orientated non-parallel to each other and to the *x,y*-axes (up to 45°). The algorithm worked properly. However, tracking the ROI at higher magnification (12kx, 20kx) presented difficulties due to the angle with the x-/y-axis. Because the iLEM stage cannot rotate, it is better to position the ribbons on ITO such that they are aligned in parallel to the Y-axis. The second sample had three ribbons and 53 sections with a curved distribution and very low quality at the boundaries: ragged profiles, corners with wedges, broken sections, and black regions of glue. The auto-navigation performance was good even without user intervention and manual correction on the detected boundaries after using Tomo’s automatic tools. Therefore, the technique can perform well with poor samples and performs best with relatively parallel ribbons.

#### Fluorescent samples

Finally, we wanted to show the versatility of our approach by using cells instead of tissue and by using fluorescence. We imaged human U2OS cells that express GFP-tagged protein condensates. These types of membraneless assemblies form via a process called protein phase separation [37]. Recently, it was shown that the Arabidopsis thaliana protein FLOE1 can phase separate in both plant and human cells [38]. We now imaged these FLOE1-1 condensates using Tomo. Figure 6d shows the result of an ROI tracked over 21 sections. As before, the LM imaging was carried out using ambient pressure, specifically as GFP fluorescence is quenched at high vacuum [21]. As a proof of concept for our approach, Fig. 6d highlights correlated fluorescent condensates in the cellular context imaged in EM and in 3D (Additional file 9: Movie S3).

## Discussion

Here, we present a novel workflow to perform fast correlated AT on a high-resolution iLEM setup in a semi-automatic manner. Our approach is best suited for efficient imaging specific structures across sections in tissue in a targeted fashion. This strategy is complementary to the slower but more complete approaches that aim to image entire sections at high resolution and create large volumes, for example, with multibeam SEM approaches [39]. Our solution is open source and can help to introduce guided navigation and acquisition more widely into AT. It leverages an iLEM to correlate the images, using the same stage to drive the microscope to regions of interest in consecutive tissue sections for both EM and LM modalities, and reconstruct correlative 3D volumes. We combine the information from LM and EM for navigation, using feedback loops whereby the available position information from lower magnifications is used for refining the locations of the structures of interest at higher magnification. This, in turn, allows us to accelerate the workflow, saving time on the navigation to the regions of interest. On top of this, the improved accuracy now allows for smaller margins around the ROIs, reducing the image size and hence recording time, and additionally, a reduced data-overhead.

In our approach, the starting point is a transmitted light image to identify the ribbons and the sections (Fig. 1, 2). In our first example, we have been using our updated Golgi staining protocol for correlation [36]. This version of Golgi staining can be imaged with transmitted LM and in EM mode. However, the Golgi stain used here can be seen as a placeholder for other immunohistochemistry precipitation-based protocols like horseradish peroxidase (HRP) and 3,3′-diaminobenzidine (DAB) and miniSOG staining [40, 41]. Consequently, we believe that the use of regular cytochemistry protocols opens new possibilities for AT. Next to Golgi and cytochemistry stained samples, the workflow presented here can be expanded easily to samples preserving fluorescence in resin (Fig. 6). Such correlative imaging using an iLEM has been carried out before [18, 42, 43]; however, such protocols that allow correlative fluorescent in resin and EM imaging are a compromise for both modalities while maintaining fluorescence and/or antigenicity within tissue samples for high-resolution SEM remains a challenge. While we are sure that future improvements in this respect may ease sample preparation, we believe that an iLEM provides an ideal starting point to perform in situ correlated AT.

The use of transmitted light overviews for navigation and stepwise imaging also promises to be the best option when using fluorescence as the second step for correlation, as the wavelength of transmitted light can be tuned to avoid bleaching; additionally, our navigation approach does not rely on SEM overview maps which can also quench the fluorescence signals. Using fluorescence for the correlation at higher magnification would also open the possibility of expanding the workflow to super-resolution imaging [21].

The detection of all sections present in the overview image is crucial, as this provides the starting point for our auto-navigation approach (Fig. 1). However, the shape of the sections can vary due to the sample preparation for the iLEM, where the original resin block containing the sample of interest is trimmed at steep angles so that the shape and dimensions of the individual sections will change gradually along the length of the ribbon. Other knife-related artifacts, such as compression of sections, may also affect the shape to some extent; in addition, the inner/outer vertex of a curved ribbon can lead to deformations, and the axis of cutting may be a bit tilted with respect to the normal vector of each plane cut. Finally, next to cutting artifacts, differences from section to section may also arise from other causes, such as the imperfect drying down of sections on the surface of the ITO-coverslip. Also, the generation of the overview image by stitching may also result in error (e.g., local deformation). Here, we used 150 nm thick sections, as this thickness gives good contrast in transmitted light and allows better than confocal resolution in the z-direction, but is not too thin to limit the number of fluorophores detected per section and so strongly affect signal to noise and contrast.

Although the quadrilateral shape used in section detection matches the shape of most sections in general, the subtle changes in the shape along the whole set of sections need to be accounted for. We considered several methods, such as binary watershed division and template matching, as well as active contours. We decided on the latter since it can deal with the small variations in shape and the sometimes irregular outlines of the sections; the automated recognition of sections along a ribbon with minimal user input using an active contours model (Fig. 3, Additional file 3: Figure S1) is thus one of our method’s key aspects and can easily be adapted to other shapes of sections than the trapezoid used here.

In addition, the finite element transformation we use for normalization (Fig. 4) is more general than the more common rigid registration as it preserves both shape and size as well as linear and angular dimensions; one of the key features provided by such a transfinite transform is the ability to link different quadrilateral geometries to the unique geometry of the reference [33].

Both the use of active contours and this finite element approach for normalization contrast with existing commercial software solutions that use a template matching approach (Additional file 10: Table S1). While some commercial solutions use a section-based coordinate system for ROIs, our normalization in connection with contour detection allows for correcting for shape changes, cutting artifacts, as well as enabling precise prediction of the position of ROIs within the section; hence, we believe this defines a new state-of-the-art.

For navigation, the vendor version of our setup uses relative coordinates. However, establishing an absolute coordinate system and saving the corrections for the auto-navigation has the advantage that the acquisition across magnifications and across ribbons can be set up easily. In addition, multiple ROIs can be parallelized, and for example, multiple LM acquisitions can be pooled before proceeding to the EM level. This can save additional time for systems where LM and EM are separated, and switching from ambient pressure (LM) to high vacuum (EM) requires pumping down the specimen chamber and evacuating the chamber needs to happen only once. The auto-navigation information for an ROI can also be recycled for another ROI that appears in the same set of sections. Likewise, for features identified at the EM level, the corresponding LM context can be checked.

The achievable accuracy for repeated correlation depends on several factors, including the positioning system’s accuracy and the lowest resolution present in the image pair used for the correlation. However, the time to acquire high-resolution maps, bleaching, and quenching by electron beams are arguments against the use of high-resolution maps for navigation. In our workflow, we try to overcome this by providing multiple intermediate steps for correlation. Also, for systems using different devices for the correlation, the imprecision of the different stages used adds up.

Consequently, to arrive at the highest level of accuracy in our approach, we use stepwise refinement by using the information from registering the images of the stack at each increase of magnification as a feedback to increase navigation accuracy (Fig. 1). This stepwise improvement helps to bridge the resolution gap between EM and LM, improving the success rate of tracing ROIs. The improved accuracy for navigation (Additional file 7: Figure S3) allows to define smaller ROIs and minimize data overheads while increasing the speed of acquisition (2.5 h for 36 aligned sections); this speed gain is a key advantage of our approach. Stepping up the resolution using registered datasets also means creating nested 3D volumes. Consequently, the images from one ROI provide either more context or more resolution in a pyramidal fashion ranging from 20x optical to 30kx EM imaging.

Generally, the larger the jump in magnification, the more challenging the prediction of the position of the ROI. This also holds true for all jumps in magnification, for both EM and LM, but is more relevant to EM due to the larger ranges of magnification available. This also means that the magnification numbers listed are typical examples, and the workflow can operate with deviations from these numbers as long as the jumps in magnifications are not too large. In fact, Fig. 6d shows that we vary between 10kx and 12kx EM magnification. A crucial step is the switch between LM and EM after the LM stack of images is acquired with the 100x lens, and the alignment of the image stacks determines the accuracy when switching to higher magnifications. Compared to EM images, the information attainable at the LM level changes to a much smaller extent, resulting in higher correlation coefficients, and hence smaller errors. This is because features distinguishable in LM are larger than those distinguishable in EM; their shape and contrast remain “constant” over more sections. For example, in LM (100x), nuclei with diameters of several μm can serve as markers for alignment of ROIs as they can be traced back in tens of sections of 150 nm. In EM, these nuclei would be far too large, and mitochondria would take their place instead, which, however, would only be re-traceable in a couple of sections, changing shape faster. While in the EM images, there is a continuous ultrastructural context, this scaling effect renders the process of alignment non- trivial. This is also the reason why there are 7 sections presented for the 30kx magnification in Fig. 6.

Overall, the traceability of structures also depends on the contrast or the staining of the involved structures. Therefore, focus adjustment to maintain the maximum contrast for each section is essential and unfocused images would lead to more errors. As the high-resolution iLEM used here requires vacuum compatible oil, which is rather viscous, we introduced a waiting time in Tomo between stage movements and acquisition of the image to allow the focus to stabilize.

When we plotted our auto-navigation’s progressive refinement in the *x,y* direction using one specific ROI along the sections, we show that we initially had to correct by up to tens of microns (Additional file 7: Figure S3). Interestingly, the error in the y-direction was slightly larger than the error in the x-direction, which could be related to the position of the sections on the ITO coverslip (straight ribbons almost parallel to the y-axis), as the movements from section to section are almost purely in *y*-direction. The trend in the magnitude of the error in navigating from one section to the next along a ribbon is fairly constant but is perturbed upon navigating from the last section of the first ribbon to the first section of the second ribbon (Additional file 7: Figure S3, from section 15 onward). This appears to be due to the long movement of the stage in the opposite direction. However, after the first section of the second ribbon, the magnitude of error in navigating to the next ribbon is comparable to the error observed in the first ribbon.

While the commercial solutions are constantly improved and developed further, the lack of accuracy of the automatic acquisition of commercial solutions has been emphasized before [27]. The official accuracy of commercial solutions is difficult to obtain but has been reported to be in the range of 5 μm. In our case, we can see from Additional file 7: Figure S3 that for the level of correlation at 100x, the accuracy is better than 5 μm. For a short summary of the general approaches used by commercial solutions, recently published tools, and our method, please see Additional file 10: Table S1.

Generally, an ITO coverslip of 22 × 22 mm can harbor 100 to 200 sections, which would require about 2-5 user interactions to image. Using our approach, we were able to follow several ROIs and reconstruct them in 3D (Fig. 6). Also, our approach is robust and operates even with suboptimal samples (Additional file 8: Figure S4). Nonetheless, it performs optimally with ribbons oriented parallel to the y-axis, which can be easily taken care of during ultramicrotomy. It is currently limited to quadrilateral sections. Autopiloting can be initiated from any spot, which is different from related publications [18, 42]. This makes our approach best suited for quantitative analysis of specific features within tissues such as cell-specific organelles, synapses, or cellular contact sites and maximizes the information obtained from a specific tissue block.

Our approach is a natural extension of “Simultaneous Correlative Scanning Electron and High-NA Fluorescence Microscopy” [16]. However, our method is different from the recent article by Burel and co-authors [27], as we base our approach on detecting ROIs in consecutive sections instead of a fluorescent guided approach for single ROIs and use of a CorrSight microscope to correlate the views between LM and EM. Dissimilar from us, Delpiano and co-workers [42] focus on “Automated detection of fluorescent cells in in-resin fluorescence sections for integrated light and electron microscopy.” Likewise, the central aspect of “Correlative super-resolution fluorescence and electron microscopy using conventional fluorescent proteins in vacuo” is not the auto-navigation from one section to the next and acquiring 3D stacks in an automated fashion. In our approach, we draw inspiration from “Micropilot: automation of fluorescence microscopy-based imaging for systems biology” [44], which was focusing on high content application in live-cell imaging and aimed to create a smart microscopy application for AT in iLEMs.

## Conclusion

We provide a proof of concept and software tools that can be used in combination with the commercial iLEM used here or standalone with other/home-built systems.

Overall, we believe that our method is ideally suited for targeted AT imaging of tissues using an iLEM setup and expect this tool to be useful for many different disciplines. We believe that our approach will be well suited for imaging, for example, of synapses and spines in neuronal tissue but also for investigating changes in stroma cells upon tumor-stroma interactions. The technique can be applied with Golgi staining, horseradish peroxidase (HRP) coupled antibodies, miniSOGs, or other traditional histological labeling techniques. Likewise, fluorescence staining or fluorescent expression proteins are possible. In addition, it is compatible with different implementations of iLEM. Given the fact that the developed workflow is an extension of the tedious manual exploration and is based on modular software tools that we make freely available, we expect quick acceptance and implementation of this novel imaging application.

## Methods

### Sample preparation

All animal experiments were approved by the KU Leuven Ethical Committee (protocol P138/2017) and were performed in accordance with the Animal Welfare Committee guidelines of the KU Leuven, Belgium. A sample from a 4–6-week-old male C57BL/6 mouse was used. Animals were euthanized with a mixture of ketamine and xylazine as per institutional guidelines.

For sample preparation, mouse brain blocks were prepared using the Golgi staining protocols for LM and EM as previously published [36]. In short, vibratome sections were incubated with 1% silver nitrate (#RT210560, EMS, USA) in dH_2_O and, consequently, cropped and incubated in 0.05% gold chloride and ice-cold 0.5% oxalic acid. Samples were then stained with 1% osmium tetroxide (#19152, EMS, USA) and 1.5% potassium ferrocyanide (#455989, Sigma-Aldrich, Belgium), followed by 0.2% tannic acid (#21700, EMS, USA), and again 1% osmium tetroxide. Next, samples were incubated in 0.5% uranyl acetate (#22400, EMS, USA) in 25% methanol overnight at 4 °C and stained en bloc with Walton’s lead aspartate [45] for 30 min at 60 °C. After washing, samples were dehydrated in ascending ethanol series and were treated with propylene oxide and flat embedded in Epon 812.

U2OS cells (ATCC, HTB-96) were grown at 37 °C in a humidified atmosphere with 5 % CO2 for 24 h in Dulbecco's Modified Eagle’s Medium (DMEM), high glucose, GlutaMAX + 10 % Fetal Bovine Serum (FBS), and pen/strep (Thermo Fisher Scientific). Cells were transiently transfected using Lipofectamine 3000 (Thermo Fisher Scientific) according to the manufacturer’s instructions. The plasmid and sequence encoding the GFP-FLOE1 protein (i.e., 8xY/F-S mutant) are described in [38].

Plated cells were lightly fixed with 2% PFA and washed 3x with PBS. The cells were scraped and pelleted at 200×*g* after which they were resuspended in 20% BSA and pelleted again at 200×*g*. The loosely packed cells in BSA were high-pressure frozen in a Leica Empact 2 high-pressure freezer (Leica, Vienna, Au), and submitted to a quick freeze-substitution protocol that preserves fluorescence [18, 46]. Briefly, frozen samples were freeze-substituted in acetone containing 0.2% uranyl acetate and 5% H2O in a Styrofoam box on a rotating platform while the temperature was allowed to rise to − 50 °C at which moment they were transferred to the Leica AFS2 automatic freeze-substitution apparatus equipped with a Leica FSP processing robot (Leica, Vienna, AU). After total time elapsed between − 80 °C and − 50 °C amounted 1.5 h, samples were washed in acetone and infiltrated in Lowicryl HM20 resin (EMS, Hatfield, PA, USA), and finally polymerized at − 50 °C by UV-light.

The samples were then trimmed in the RMC Powertome PC Ultramicrotome, in a trapezium shape with a trimming knife (#DUTB30, laborimpex) for straight edges. On the bottom and the upper edge of the trapezium, glue was added to obtain a rigid ribbon; the glue is a mix of 1:1 dap Weldwood original contact cement (#B0006MXRWU, Amazon): xylene (#1.08298.4000, VWR) applied with a paintbrush and dried for 2 min. Section cutting started with 150 nm sections with an ultra ATS DiATOME diamond knife (#30-UL-ATS, DiATOME U.S.). Ribbons were collected on conductive ITO coverslips of 22 × 22 mm by removing the water from the diamond knife. Coverslips are then mounted using an aluminum sample holder stuck with adhesive tape. The sample holder has a central hole of 19 mm, which determines the serviceable surface where the ribbons can be imaged.

### Data acquisition

For image acquisition, a SECOM platform from DELMIC B.V. (Delft, the Netherlands) was used. The SECOM was mounted on a JEOL JSM 7200F LV (Tokyo, Japan) Scanning Electron Microscope. For the LM image acquisition, a Nikon Plan Fluor 20x with a NA of 0.45 and a Plan Apo VC 100x oil immersion objective lens with a NA of 1.4 were used. For immersion, Delmic’s high vacuum compatible oil was used. Transmitted light illumination was realized with a home build LED ring illumination and a diffuser plate mounted above the sample stage (see Fig. 2). For LM acquisition, an Andor Zyla sCMOS camera was used. For sample movement, a Mercury-II stage from PI was integrated into the Odemis software. The LM and EM images are aligned according to the manufacturer’s description.

For EM imaging, tuning of the EM parameters was done for each sample before starting the EM automatic acquisition. Concerning EM tuning parameters, the best results were achieved with ribbons consisting of 150 nm thick sections where focus, brightness, contrast, and astigmatism were manually tuned; additionally, autofocus and automatic astigmatism corrections were invoked from the software of the EM, however, less robustly with increasing magnification. Back-scattered electron images were acquired at 3 kV accelerating voltage.

### Data processing

For information about the data processing steps, as well as the environment and how to use it, please see the supplementary Information Technical Documentation and supplementary Information User Manual. For a short overview, please see below.

### Stitching

For the stitching of the navigational overview images, we used the Microscopy Image Stitching Tool (MIST) or Grid/Collection stitching, which are readily available in Fiji/ImageJ [29–31]. We used an overlap of 20% for the images.

### Ribbon recognition

For the ribbon recognition, standard image processing tools like Denoising, Laplacian blur, and thresholding have been made available in a custom-made tool that allows stepwise to repeat and combine the individual processing steps with the goal to create images that can be easily detected by active contours. For more information, please see the supplementary Information User Manual. The preprocessed image can be saved for later.

### Active contour

After preprocessing the overview image, the sections can be detected with an active contour algorithm implemented in our custom-made “Tomo” tool with minimal user guidance. The following steps include detecting the sections as polygons and optimizing the detection. This process can then be expanded to complete ribbons. For more information on the implemented active contour algorithm, please see supplementary Information Technical Documentation.

### Focus map acquisition

Before starting the image acquisition, the user may manually focus the microscope at a limited number of stage positions, and natural neighbor interpolation is used to build a focus map for the full sample. During actual image acquisition, this map is consulted to obtain an accurate focus for the region of interest in every section.

### Section standardization

The polygons that are a result of the section detection can be used for normalization. By geometric transformation, the position of a region of interest in a section can be predicted even in the presence of distortions and the gradual shape change of the tissue block (see Fig. 4)

### Automated navigating to ROIs across multiple sections

The position of the sections on the navigational overview image, together with the position prediction based on section standardization, is used to move the stage for automated acquisition at higher magnifications. A focus map can be added.

### Iterative refinement

The ROI position predictions are then refined in a feedback loop using image registration of the acquired image stacks. The SIFT and StackReg image registration plugins [34, 35] available in Fiji/ImageJ [28] worked well in our hands. Montaging the images in the figures was done using ec-CLEM [11].

### Creation of test images

To test the quality of detection, existing images of ribbons were modified with noise and by removing corners from the sections to test the analysis workflow (see Additional file 4: Figure S2).

## Supplementary Information

**Additional file 1: Text S1.** Technical documentation

**Additional file 2: Text S2.** User manual of Tomo

**Additional file 3: Figure S1.** Image processing steps used to detect sections. From left-to-right, the subsequent steps in the processing pipeline are shown. **a** Close-up of the unprocessed light microscope overview image. **b** Image after contrast normalization. **c** Noise reduction via Gaussian blurring and **d** a Laplacian filter are applied. **e** Approximate outline of a sample section roughly sketched by the user. **f** Outline after active contours optimization. The resulting contour matches the true section outline nicely. Scale bar is 100 μm.

**Additional file 4: Figure S2.** Section detection tested with intentionally degraded images. **a** Modified images. From left to right: ground truth original overview image, added salt and pepper noise, erased corners and borders, (GN 10) added Gaussian noise σ=10, (GN 40) added Gaussian noise σ=40, (GN 70). **b** Whisker box plot of the tested images with added noise and removed corners and borders. Errors of the boarders of the sections of the first ribbon (see Fig. 3) are shown. Scale bar 200 μm.

**Additional file 5: Movie S1.** Movie accompanying Figure 6b

**Additional file 6: Movie S2.** Movie accompanying Figure 6c

**Additional file 7: Figure S3.** Comparison of displacement errors with increasing magnification. **a** Displacement error in x [μm] and **b** in y [μm] at each section for slice mapping on the 20x overview image and after LM acquisition with 100x lens and 4kx EM acquisition, compared to corrected navigation after 12kx EM acquisition. **c** and **d** showing the respective accumulated error of **a** and **b**. Representative data is shown.

**Additional file 8: Figure S4.** Effect of poor sample preparation on Tomo performance. **a** Ribbons with a good quality boundary but inclined distribution on the ITO coverslip. **b** Curved ribbons with multiple defects. **c** Detail from **b**, indicating glue contamination in black and additional wedges on the top right side of the sections causing deviation from the desired trapezoidal /quadrilateral shape. Scale bar **a**, **b**= 500 μm, **c** 250 μm.

**Additional file 9: Movie S3.** Movie accompanying Figure 6d

**Additional file 10: Table S1.** Comparison of Tomo features with other state of the art software solutions. Tomo, MAPS, Atlas, Mosaic Planner, and Wafer Mapper are compared.

## Data Availability

All data generated or analyzed during this study are included in this published article, its supplementary information files, and publicly available repositories. For data, please see Figshare repository; 10.6084/m9.figshare.c.5459541. The source code is available via this Github repository: https://github.com/vibbits/tomo and via the Zenodo public repository with the following doi: 10.5281/zenodo.4911607.
