# Supplementary material for: A workflow for streamlined acquisition and correlation of serial regions of interest in array tomography
Source: BMC Biol. 2021 Jul 30;19:152. doi: 10.1186/s12915-021-01072-7 (PMC8323292; doi:10.1186/s12915-021-01072-7)
Supplement: Supplementary file 1 — Additional file 1: Text S1. Technical documentation [file 12915_2021_1072_MOESM1_ESM.pdf]

Supplementary file 1: Technical Documentation

Contents

S1. Active contours for accurate section outline detection 1

S2. Preprocessing the overview image for active contours 4

S3. Region-of-interest tracking via quadrilateral normalization 5

S4. Tomo implementation 9

S1. Active contours for accurate section outline detection

Introduction

For the automated tracking of regions of interest across multiple consecutive sample sections, we need to predict an approximate position of the point of interest solely based on its position relative to the contours of the sections in which it is located. This is discussed qualitatively in the main text of the current manuscript, and described in more detail mathematically in Section S3 of this supplementary material.

The accuracy of predicting the regions-of-interest position depends to a large extent on the accuracy of the detection of the section shape, meaning by which the quadrilateral representations of the sections coincide with the true section contours.

In an ideal scenario the section detection would be fully automated. However, to be more flexible and comply with real world samples, we developed a semi-automatic approach, where the user manually draws approximate quadrilateral outlines for a small number of sections using a simple interactive drawing tool, and our software turns these single sections into accurate quadrilateral representations of ribbons of sections. Our method for generating a ribbon of section outlines is discussed in Section S4 of this supplementary material. Here we will discuss our approach to improve a rough quadrilateral outline that was drawn quickly by the user and overlaps only partially with the section boundaries, into a quadrilateral that matches the section boundaries accurately. It is inspired by active contours methods used for image segmentation.

Method description

Similar to other active contour methods (Kass M, Witkin A, Terzopoulos D. Snakes: Active contour models. International Journal of Computer Vision. 1988), ours also takes as input an image and an initial contour that serves as an approximation or starting point for the desired segmentation. The input image is the (transmitted light) light microscopy overview image with the ribbons of sample sections. The initial approximate contour is a quadrilateral, either drawn manually by the user, or

predicted by the software. The desired segmentation is a quadrilateral that matches the true outline of the section.

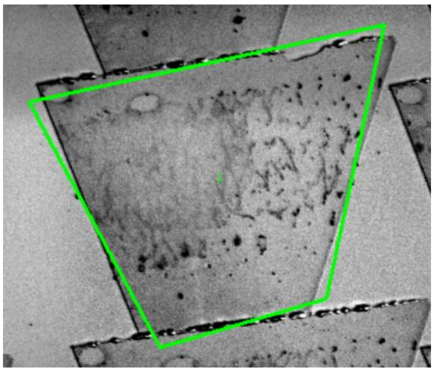

Figure 1 Overview image with an initial contour in green. The contour is a rough quadrilateral approximation of the sample section's true location. The active contour method will "snap" this contour to the section outline.

One aspect where our approach differs from typical active contour methods is that normally the active contour has a relatively large number of vertices so that it can detect objects with a complicated outline. Since by design we only want to detect quadrilaterals, our active contour has just 4 points.

During our experiments we observed that the visibility of the edges of the sample sections in the overview image is sensitive to the lighting setup during image acquisition, as well as to the specific sample preparation protocol. To increase robustness, we do not perform the active contours algorithm on the raw overview image itself, but on a preprocessed version where edges are enhanced. Additionally, these edges are low-pass filtered to spread them out, which significantly increases the capture range for the active contour. We discuss the various overview image preprocessing steps in detail in Section S2 of the supplementary material.

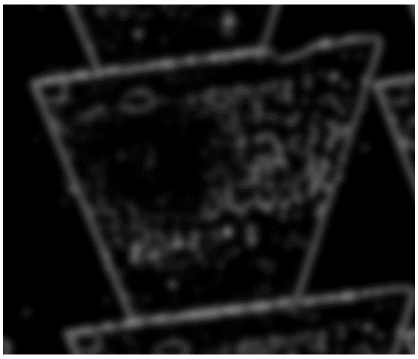

Figure 2 Overview image pre-processed for the active contours algorithm. Section edges are enhanced and spread out to increase the capture range for the active contour.

We can now formalize our active contours algorithm as follows. The active contour is a quadrilateral $\mathbf{r}_0\mathbf{r}_1\mathbf{r}_2\mathbf{r}_3$, with edges $\mathbf{r}_i\mathbf{r}_{i+1}$ and vertices $\mathbf{r}_i=(x_i, y_i)$, where i=0…3 and $\mathbf{r}_4=\mathbf{r}_0$. The (x, y) coordinates of the vertices are in image coordinates. The image in which the active contour moves is $\tilde{I}(r)$, which is the pre-processed version of the original overview image $I(r)$. High pixel values in $\tilde{I}(r)$ typically occur for edge pixels, so for pixels on the section's contour, whereas low pixel values tend to correspond to the image background.

The total energy E of the active contour $\mathbf{r}_0\mathbf{r}_1\mathbf{r}_2\mathbf{r}_3$ is defined to be the sum of the energies of its four edges $r_i r_{i+1}$:

$$E = \sum_{i=0}^{i=3} E_{r_i r_{i+1}}$$

The energy for each edge $r_i r_{i+1}$ is simply the line integral along that edge of the preprocessed overview image.

$$E_{r_i r_{i+1}} = \int_0^1 \tilde{I}(t\, r_i + (1-t) r_{i+1}) dt$$

Hence, the energy serves as a score indicating how well the active contour overlaps with the section's edges. To drive the active contour from its initial inaccurate position towards the true section outline we move the active contour vertices towards a nearby (possibly local) maximum in the energy landscape. This can be performed iteratively via gradient ascent. It is of note that the energy function needs information about relatively distant edges (so edges need to be blurred during pre-processing), and that denoising is helpful (to discard debris which otherwise may contribute to unwanted local maxima).

To implement the gradient ascent algorithm, we view the energy function E as a function of the 8 position variables of the active contour: $E(x_1, y_1, x_2, y_2, x_3, y_3, x_4, y_4)$. The gradient of E, which is required to update the position of the contour, is approximated numerically. For example $dE/dy_2 = (E(x_1, y_1, x_2, y_2+h, x_3, y_3, x_4, y_4) - E(x_1, y_1, x_2, y_2, x_3, y_3, x_4, y_4)) / h$ for a small displacement *h* of a vertex along the y direction. More accurate numerical approximations of the derivative are possible but will require more evaluations of the energy function E.

The line integrals $E_{r_i r_{i+1}}$ along the active contour edges can be approximated through sampling of the image $\tilde{I}(r)$ at equidistant positions along the edges.

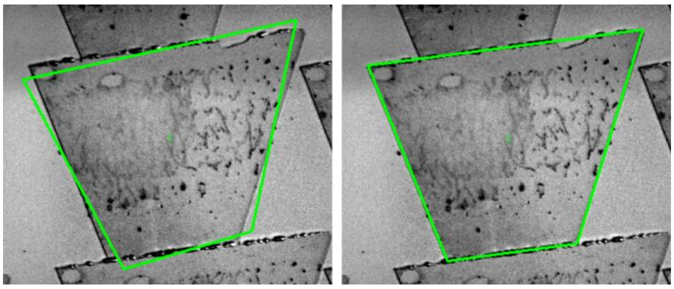

Figure 3 Active contour algorithm for finding section outlines. On the left the original section outline that was manually drawn by the user is shown; on the right the section outline after optimization with the active contour algorithm. The optimized contour matches the true outline nicely.

S2. Preprocessing the overview image for active contours

The active contours method (see Section S1 in this suppl. mat.) iteratively moves the active contour's vertex positions towards the true sample section outline in a given image. Ideally one would simply use the original transmitted light microscopy overview image of the ribbons with sample sections. This however does not work, and instead we use a preprocessed version of this overview image with the active contours algorithm. Several pre-processing steps are chained together. This is summarized in Figure 4.

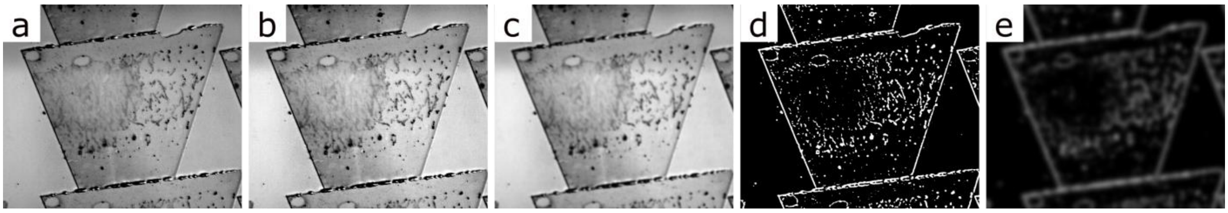

Figure 4 Image processing steps applied to the overview image in preparation of active contours. From left-to-right the subsequent steps in the processing pipeline are shown. (a) close-up of the unprocessed light microscope overview image, (b) the close-up after contrast normalization was applied, (c) after noise reduction via Gaussian blur, (d) after applying the Laplacian, and the final result (e) after applying a Gaussian blur on the thresholded Laplacian-of-Gaussian.

We will now describe the individual preprocessing steps in more detail and provide the rationale for why they are needed.

Step 1: Contrast enhancement

The first preprocessing step applied to the raw overview image is contrast enhancement. We stretch the histogram of the original overview image between the 2nd and 98th percentile so it uses the full range of possible pixel values for the image's bit depth. This normalization reduces the impact of different lighting conditions during overview image acquisition on the performance of the active contours algorithm.

Step 2: Laplacian of Gaussian

Since we want to discover the contours of the sample sections, we will enhance the section edges with an edge detection image processing step. We chose the Laplacian of Gaussian. The Gaussian blur reduces the impact of image noise and small debris or dust particles on the Laplacian edge detector (which is very sensitive to noise). This in turn reduces the likelihood for the active contour to get stuck in a local maximum formed by spurious edges.

$$LoG(x,y) = -\frac{1}{\pi\sigma^4}\left[1 - \frac{x^2+y^2}{2\sigma^2}\right]e^{-\frac{x^2+y^2}{2\sigma^2}}$$

Step 3: Thresholding

This step aims to isolate true edges pixels from spurious edge pixels. Pixel values in the Laplacian of Gaussian image that are lower than a user-specified intensity threshold T are deemed to be actual edge pixels and are mapped onto maximum intensity pixels in the output image ("white", which we

use to indicate edges for the gradient ascent). Pixel values in the Laplacian of Gaussian that are larger than the threshold T are assumed to be background pixels and are mapped to 0 (“black”).

Step 4: Low-pass filtering the edge image

The active contour method is guided by changes in the energy function, and our energy function is based directly on edge pixel intensities. Hence we must ensure that the energy landscape of the preprocessed image in the neighborhood of the true section contour varies smoothly (so that meaningful derivatives can be calculated for gradient ascent). We must also make sure that changes in the energy are still noticable at a reasonable distance away from the true section outlines, because otherwise the edges cannot “attract” the active contour.

We accomplish both goals by low-pass filtering the thresholded edges image with a Gaussian blur kernel

$$G(x,y) = -\frac{1}{2\pi\sigma^2} e^{-\frac{x^2+y^2}{2\sigma^2}}$$

S3. Region-of-interest tracking via quadrilateral normalization

To track a user specified region-of-interest (ROI) in one section across multiple consecutive sections we need to be able to predict the ROI position relative to the outline of each of those sections. The approach we propose assumes that the sections are quadrilateral in shape. It allows for small variations in shape of the sections (due to sample cutting artefacts) and in size (since the sections are often cut from a sample that is pyramidal in shape). When viewed under a microscope the section contours are obviously not exact geometrical quadrilaterals, but we further assume that they were approximated as a quadrilateral that was either drawn manually by the user, or calculated using our active-contours approach (see suppl. mat. S1).

The mathematical method that we propose is a transfinite transformation with roots in finite-element-theory. It is an invertible affine transformation that maps between an arbitrary quadrilateral and a normalized square.

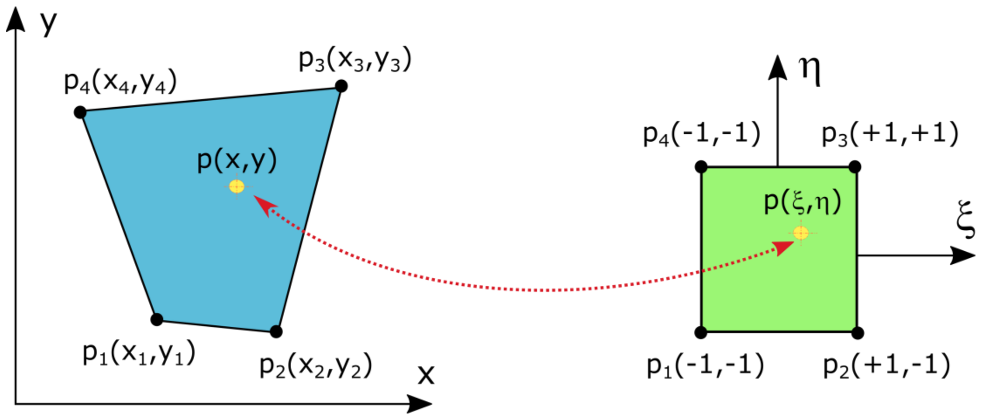

Figure 5 Transfinite transformation. This transformation is an invertible mapping between the points in a general quadrilateral (shown on the left) specified in physical coordinates (x, y), and a reference square (shown on the right) specified in natural coordinates (ξ, η). Shown in yellow is a point-of-interest p.

The quadrilateral has vertices p_1, p_2, p_3, p_4 with coordinates (x_1, y_1), (x_2, y_2), (x_3, y_3), (x_4, y_4), and region-of-interest point p with coordinates (x, y). These coordinates are "physical coordinates", for example, the microscope stage positions where these points are on the optical axis. The quadrilateral will be mapped onto a reference square in so-called "natural coordinates" (ξ, η). This square is centered on the origin and has sides with length 2. The corresponding point of interest p has coordinates (ξ, η).

Mathematically, the mapping between physical coordinates (x, y) and natural coordinates (ξ, η) is governed by the following relations:

$$\begin{cases} x = \sum_{k=1}^{4} N_k(\xi,\eta)\, x_k \\ y = \sum_{k=1}^{4} N_k(\xi,\eta)\, y_k \end{cases} \tag{1}$$

with

$$\begin{cases} N_1(\xi,\eta) = \frac{1}{4}(1-\xi)\,(1-\eta) \\ N_2(\xi,\eta) = \frac{1}{4}(1+\xi)\,(1-\eta) \\ N_3(\xi,\eta) = \frac{1}{4}(1+\xi)\,(1+\eta) \\ N_4(\xi,\eta) = \frac{1}{4}(1-\xi)\,(1+\eta) \end{cases} \tag{2}$$

The task of predicting the position of a point of interest p in section i+1 given its position in section i can now be viewed as a two-step process:

- step 1: transforming the physical coordinates of point of interest $p^{(i)}$ with known coordinates ($x^{(i)}$,$y^{(i)}$) in section i, which has known quadrilateral outline $p_1^{(i)}p_2^{(i)}p_3^{(i)}p_4^{(i)}$, to natural coordinates (ξ, η); and
- step 2: transforming these natural coordinates (ξ, η) to physical coordinates ($x^{(i+1)}$,$y^{(i+1)}$) in the next section i+1, which has known quadrilateral outline $p_1^{(i+1)}p_2^{(i+1)}p_3^{(i+1)}p_4^{(i+1)}$.

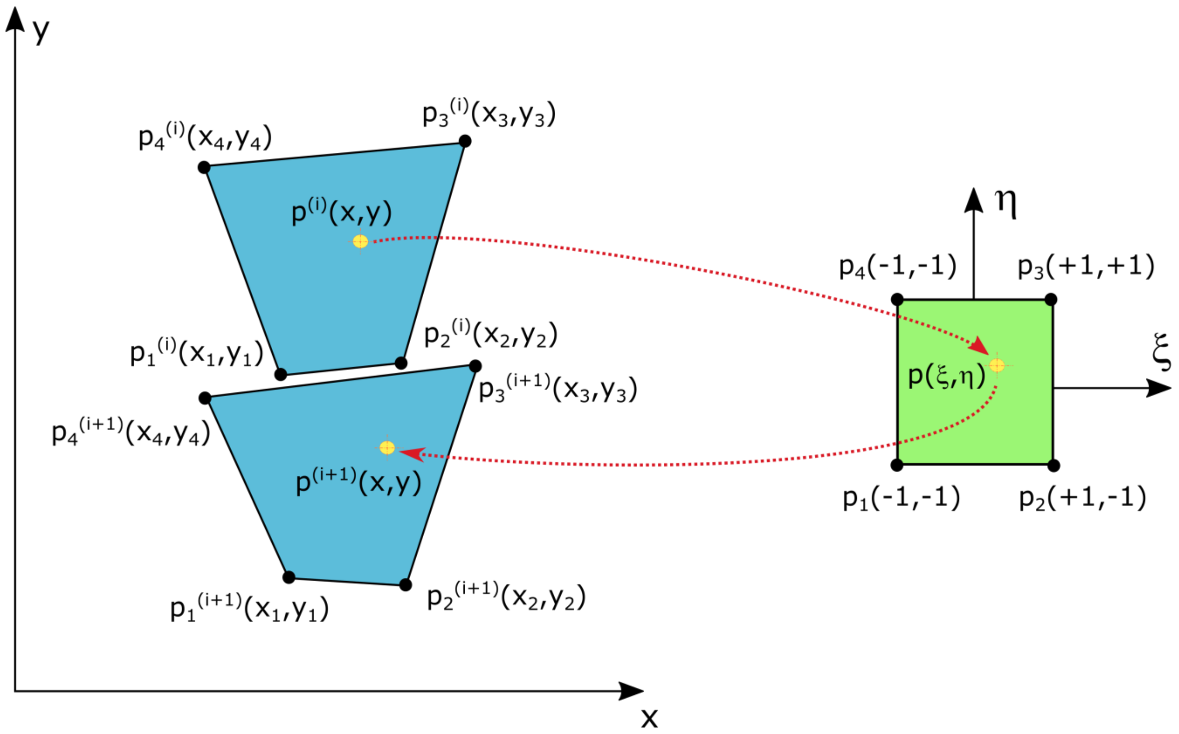

Figure 6 Two-step approach for ROI tracking. The point-of-interest $p^{(i)}$ in quadrilateral section i (top left) is first normalized to natural coordinates (square on the right) and then mapped into a corresponding position in quadrilateral section i+1 (bottom left)

In practice, the calculations for step two are trivial: plug the natural coordinates (ξ, η) of the point of interest into equations (1) and (2) to obtain corresponding physical coordinates (x,y).

To perform step one we need to invert the non-linear system of equations (1) and (2) so we can calculate natural coordinates (ξ, η) given physical coordinates (x, y).

We omit the full algebraic manipulations here but provide the resulting solution (ξ, η) for completeness:

$$\eta = \frac{-b \pm \sqrt{b^2 - 4ac}}{2a} \qquad (3)$$

where

$$\begin{aligned} a &= E*C - B*F \\ b &= F*G - H*C - B*D + A*E \\ c &= G*D - A*H \end{aligned}$$

and

$$
\begin{aligned}
A &= -x_1 + x_2 + x_3 - x_4 \\
B &= -x_1 - x_2 + x_3 + x_4 \\
C &= x_1 - x_2 + x_3 - x_4 \\
D &= -y_1 + y_2 + y_3 - y_4 \\
E &= -y_1 - y_2 + y_3 + y_4 \\
F &= y_1 - y_2 + y_3 - y_4 \\
G &= 4x - (x_1 + x_2 + x_3 + x_4) \\
H &= 4y - (y_1 + y_2 + y_3 + y_4)
\end{aligned}
$$

To ensure that we map into the reference square, the sign for the square root in equation (3) must be selected such that $-1 \leq \eta \leq +1$.

And finally

$$\xi = \frac{G - B * \eta}{A + C * \eta}$$

S4. Tomo implementation

We implemented a proof-of-concept of our approach for semi-automated array-tomography that is described in the paper and we made it available as open-source. This software application is called "Tomo". The source code is available in this Github repository: https://github.com/vibbits/tomo. Tomo is an interactive stand-alone application with a graphical user interface (GUI) and is implemented in Python. Since Tomo is designed to facilitate array tomography on the SECOM platform, it necessarily needs to interact with the Odemis control software for the SECOM. This requirement dictated several design decisions for Tomo. Since Odemis runs on Ubuntu Linux, so does Tomo. Tomo also uses the same Python modules that Odemis is built on. This decision however leaves the door open to merge all or parts of Tomo into Odemis itself should this be desirable at a later time.

In general, we tried to leverage existing open-source software libraries and solutions as much as possible.

The GUI for Tomo is implemented in wxPython, and it uses a NavCanvas to display the overview image and all graphics elements such as sample section outlines in a graphical canvas that can be zoomed and panned.

We use OpenCV and scikit-image for image manipulations, and NumPy for numeric calculations.

For registration of the images of the region-of-interest in a series of successive sections, Tomo runs a Fiji instance in the background. This instance in turn calls a Fiji plugin to perform the registration. For registration Tomo supports both "Linear Stack Alignment with SIFT" and "StackReg". A simple private modification (available upon request) was done to these two Fiji plugins to also output the transformation matrices used to align the images with one another. Tomo parses this output and uses the transformation matrices to calculate improved region-of-interest predictions.

For focus interpolation from a limited number of focus points set by the user, is done via so-called natural neighbor interpolation. We used an existing open-source Python package (https://github.com/innolitics/natural-neighbor-interpolation) that implements a discrete version of Sibson's method for natural-neighbor interpolation. It can efficiently generate a dense 2D grid of interpolated values.
