# Supplementary material for: A workflow for streamlined acquisition and correlation of serial regions of interest in array tomography
Source: BMC Biol. 2021 Jul 30;19:152. doi: 10.1186/s12915-021-01072-7 (PMC8323292; doi:10.1186/s12915-021-01072-7)
Supplement: Supplementary file 2 — Additional file 2: Text S2. User manual of Tomo [file 12915_2021_1072_MOESM2_ESM.pdf]

Tomo v1.2 User Manual

Contents

Introduction........2

Installing Tomo........2

Starting Tomo........2

Tomo user interface overview........2

Preferences........3

Acquiring the overview image........4

Loading the overview image........4

Exploring the overview image........7

Identifying section contours on the sample........7

Manually drawing the section outlines........8

Selecting and editing section outlines........9

Extending a section outline into a ribbon of sections........9

Active contours for outline improvement and ribbon building........11

Aligning microscope stage and overview image........15

Building a focus map........16

Specifying the region-of-interest........19

Light microscope image acquisition........20

Electron microscope image acquisition........23

Introduction

Tomo is an interactive, graphical software application that implements a semi-automated workflow for array tomography on the SECOM platform. It is designed to work in close cooperation with Odemis.

Odemis is the control software for the SECOM, a platform for integrated light microscopy (LM) and electron microscopy (EM), commercialized by DELMIC B.V. (Delft, The Netherlands). In our case the SECOM platform is retrofitted to a Jeol SJF720 Scanning Electron Microscope (JEOL Ltd., Tokyo, Japan).

Odemis controls the LM and EM microscopes, performing such tasks as microscope stage movement, setting the imaging parameters, focus position, and doing the actual LM and EM image acquisition. Tomo on the other hand drives the higher level automation pipeline that triggers the lower level tasks that Odemis then takes care of.

This document will guide the user through the different steps to be followed in Tomo and Odemis, for performing the automated array tomography pipeline we describe in the accompanying paper.

Installing Tomo

Please follow the detailed installation instructions on the GitHub page for Tomo: https://github.com/vibbits/tomo

Starting Tomo

Open a terminal window on the computer that also runs Odemis. Then change to the directory that Tomo was cloned into (via git), or extracted into (from the zip archive on GitHub). For example, if the Tomo Python sources are present in the "~/tomo/src" then do a "cd ~/tomo/src". Finally, start Tomo via "python tomo.py".

The Tomo window with its graphical user interface (GUI) will appear.

Tomo user interface overview

When Tomo is running, its application icon is present in Ubuntu's Launcher at the left-hand side of the screen:

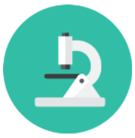

The Tomo user interface consists of a menu bar (which on Ubuntu may be on top of the screen, separate from the Tomo window), a tool bar with icons for the different interactive tools, and a large canvas.

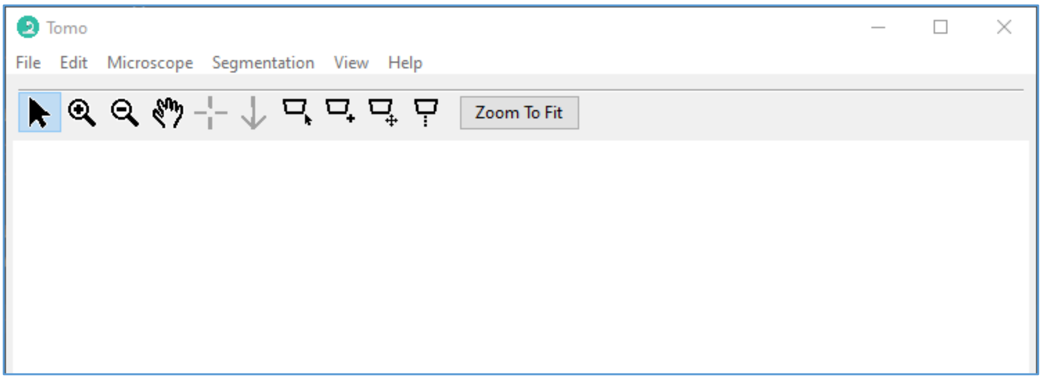

The canvas below the tool bar is initially empty, but will be used for displaying the overview image, the sample section outlines, the region of interest, etc.

The table below lists the different tools in the tool bar. Their functionality will be explained in more detail in later sections of this document.

	Selection
	Zoom in
	Zoom out
	Pan
	Mark
	Move stage
	Polygon selection
	Polygon creation
	Polygon editing
	Ribbon builder
Zoom To Fit	Zoom to fit selection rectangle

Preferences

When using Tomo for the first time, we have to point it to a few external tools that it uses: the location of Fiji (for LM and EM image registration), the Odemis command line tool (for controlling the stage), and the script that will be passed to Fiji to perform image registration. In case these tools are not installed in default locations, their paths can be specified in the Preferences dialog (Edit > Preferences…).

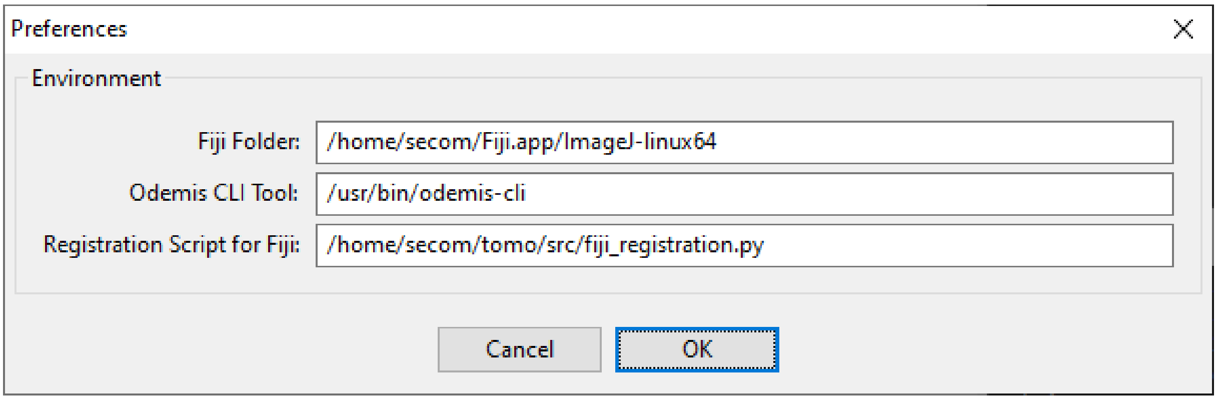

We are now all set to use Tomo.

Acquiring the overview image

The basis for our proposed automated tomography pipeline is a low magnification, but large field-of-view light microscope (LM) overview image of the sample holder with the sample sections. This overview image will allow us to specify the approximate position of our region-of-interest on one section, and will then guide later, higher magnification LM and EM image acquisitions of this region-of-interest on many successive sections.

The overview image is acquired as a grid of overlapping image tiles, using an existing plugin[1] for Odemis. This plugin allows the user to specify the number of rows and columns in the grid, as well as the amount of tile overlap, and then performs an automated image acquisition.

These image tiles are then stitched into a single large composite overview image. Stitching can be performed using any available tool, for example the MIST[2], or the Grid/Collection Stitching[3] plugins in Fiji.

Loading the overview image

The stitched LM overview image can now be imported in Tomo via File > Import Overview Image…

In the import dialog, we can select the overview image via the Browse button, or enter the exact path in the edit field. (Tip: here and in many other dialogs in Tomo, the edit fields will remember their most recent value. So the next time this dialog appears the initial values in the edit fields will be the ones the user entered the previous time.)

[1] Its description is outside the scope of this document.
[2] https://pages.nist.gov/MIST/
[3] https://imagej.net/Grid/Collection_Stitching_Plugin

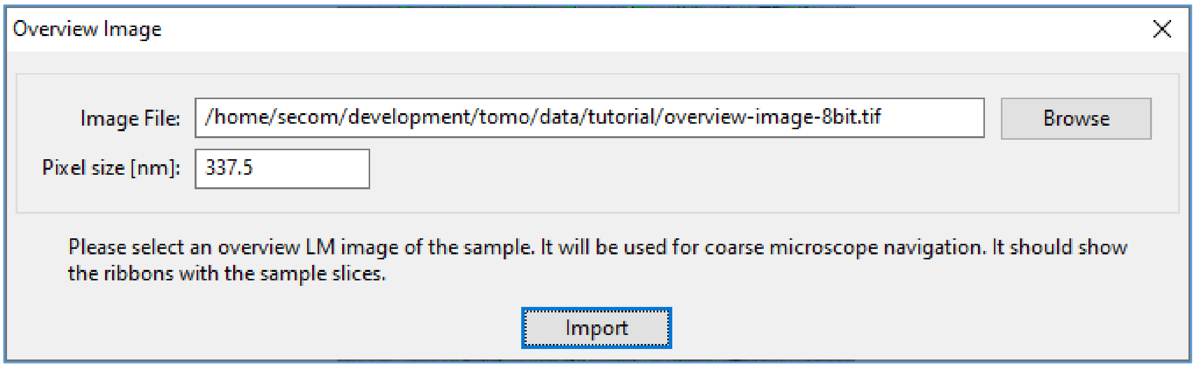

We also need to specify the size of the pixels in the overview image (in nanometer). This knowledge will allow Tomo later to convert distances measured in pixels on the overview image to physical distances between points on the sample.

In our case, the objective lens used for the image tile acquisition is a 20x dry lens (Nikon S Plan Fluor 20X, NA 0.45, WD 8.2-6.9 mm). This results in a pixel size of 337.5 nm.

After pressing Import, the overview image appears in Tomo's canvas:

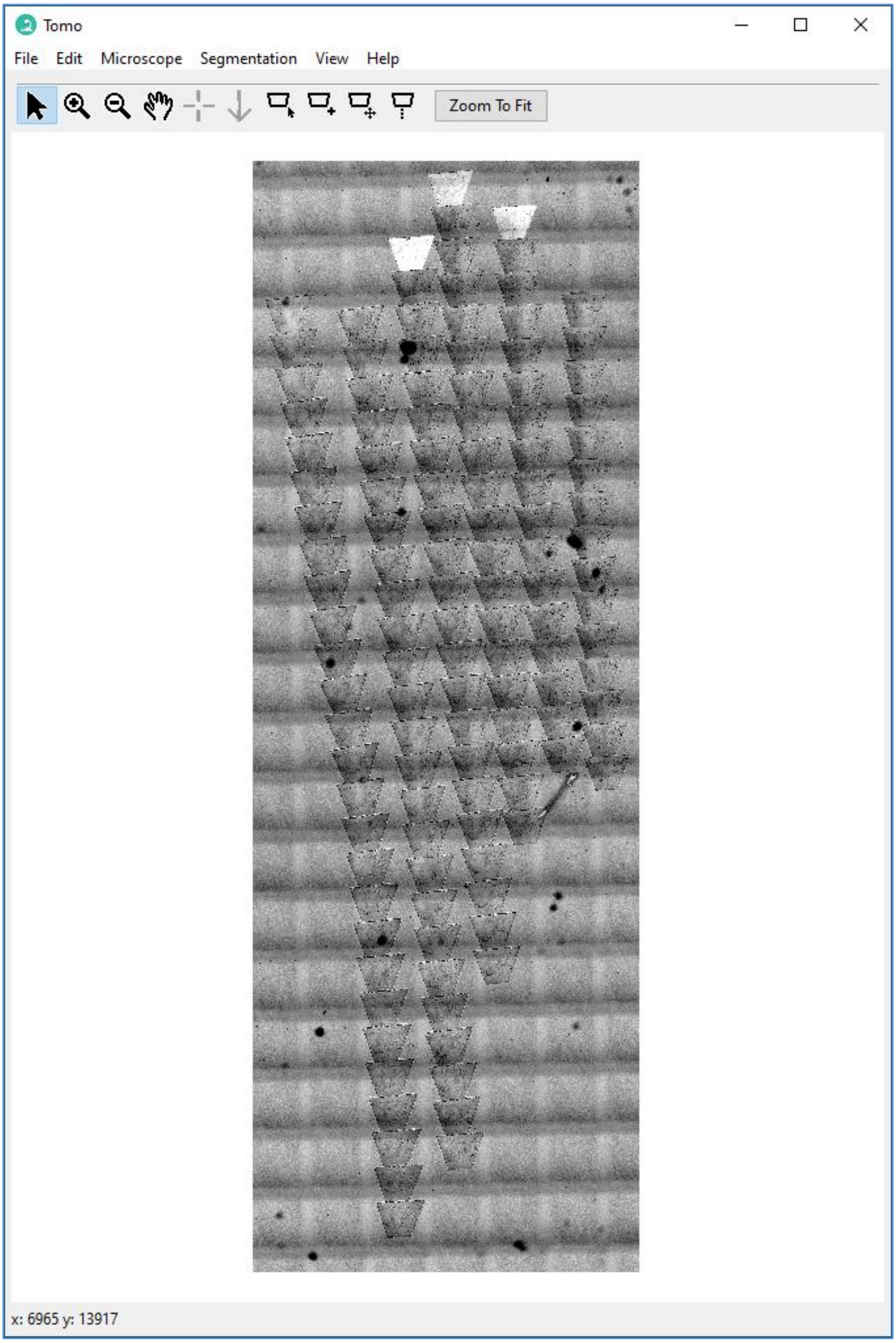

Exploring the overview image

The canvas in Tomo offers a number of tools for exploring the overview image:

The Pointer tool

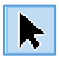

If this tool is selected, the cursor acts as a simple pointer. Underneath the overview image, the (x, y) coordinates are shown of the pixel of the overview image that the Pointer is over. This feature is mostly for debugging purposes.

Zoom out tool

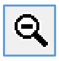

Select this tool and click on the overview image to zoom out.

Zoom in tool

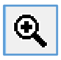

Select this tool and click on the overview image to zoom in.

Alternatively, one can also click and drag a rectangle on the overview image, to zoom in on that particular portion of the image.

Zoom to fit button

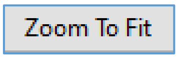

Click the Zoom To Fit button to automatically zoom in/out such that the complete overview image fits inside the Tomo window.

Pan tool

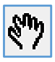

Select this tool, then click and drag the overview image to move it around in the Tomo window. This is useful if we have zoomed in on the overview image and it is too large to be shown fully.

Identifying section contours on the sample

To be able to track a region-of-interest across several consecutive sections, we need to specify where these sections are on the overview image. Tomo assumes that the sections are quadrilateral in shape, so their position is uniquely determined if the 4 corners of their quadrilateral outline is specified.

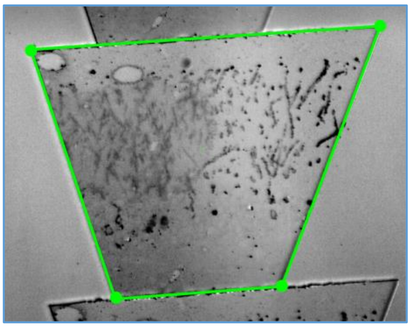

Please note that in the current version of Tomo, the sections must be specified in the correct sequential order: imaging and creation of a 3D stack of these images afterwards will happen in the order in which the section outlines were specified[4] in Tomo.

Tomo offers four tools to let the user specify the section outlines easily:

1. outlines can be drawn manually on the overview image using an interactive tool;
2. existing outlines can be edited interactively;
3. an existing outline can be duplicated interactively into a ribbon of outlines;
4. a roughly drawn *inaccurate* outline can be optimized automatically to an outline that *accurately* matches the section contour.

Since these section outlines are critical for the automated array tomography pipeline we envision, after creating the outlines, we will save them for later via File > Save Slice Polygons… Similarly, we can also load a set of section outlines that were saved before via File > Load Slice Polygons.

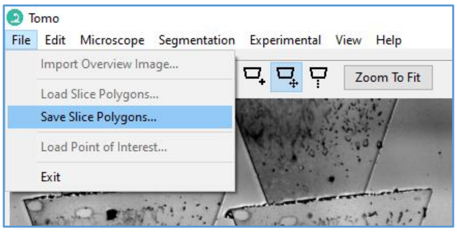

We will now discuss the different ways to create the section outlines in full detail.

Manually drawing the section outlines

We start by looking at the overview image to find that sample section which was cut *first* from the sample, and will manually draw its quadrilateral outline.

We select the Polygon Creation tool

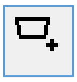

[4] A future version of Tomo will allow the user to draw the sections in any order, and an interactive tool will be added to Tomo that will allow the user to impose or modify the sequential order in which the sections were cut from the sample afterwards.

from the tool bar and left click on each of the 4 corners of the section in turn. The figure below shows the process in action. After each mouse click an extra point is added to the section contour, and after the fourth click Tomo ends the polygon creation for that section.

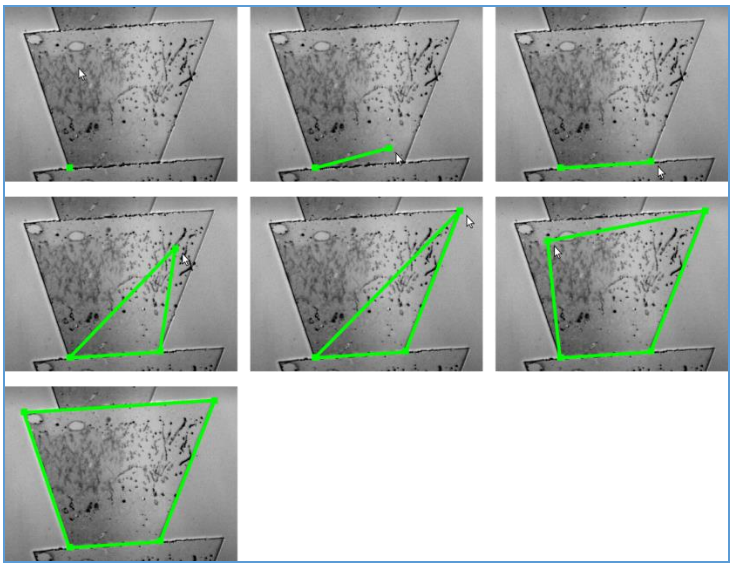

Note that the order in which the section corners are specified matters: it must be the same for each of the sections, for example by always starting with the bottom left corner of the section, and specifying the corners in counter-clockwise order. This allows Tomo to identify corresponding corners in any two sections, which is important for some operations later on.

Selecting and editing section outlines

Outlines in Tomo can be in the selected or unselected state. This is indicated by the line thickness of the outline: thick lines for selected outlines, thin lines for unselected ones.

Outlines can be selected using the Polygon Selection tool:

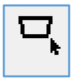

With this tool active, click and drag a rectangular selection around the sections that need to be selected. The outlines will become thick to indicate they are selected. It is also possible to select *all* outlines by pressing Ctrl-A.

Selected outlines can be edited using the Polygon Editing tool:

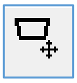

With this tool active, move the mouse cursor over a corner of a selected outline. The corner's rectangular handle will turn red to indicate that it becomes active. The handle can now be dragged around to change the shape of the section outline.

If desired, selected outlines can be deleted by pressing the DEL key.

Extending a section outline into a ribbon of sections

Most sample sections are not present as individual isolated sections, but rather as ribbons of sections that are connected via a common border. While it would possible to draw each section manually using

the Polygon Creation tool, in the case of ribbons it is advantageous to duplicate an existing section outline into a long strip of identical sections.

This goes as follows. First select a template section outline using the Polygon Selection tool. Then switch to the Ribbon Builder tool:

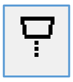

Now move the cursor over the bottom left corner of the section outline, then click and drag this corner. While dragging, Tomo will continuously estimate the number and the position of sections that is needed to close the gap between the template section and the current mouse position.

As we move the cursor around, this suggested strip op new sections is updated and shown in real-time with dotted lines on the overview image. Move the cursor over the last section of the ribbon on the overview image, such that the predicted sections and the true ribbon overlap as good as possible, and release the mouse button to create a full strip of section outlines.

This process is depicted visually in the series of six images below. The first image shows the template section polygon, then four intermediate ribbons are shown as the user moved the cursor around, and the last image shows the completed ribbon.

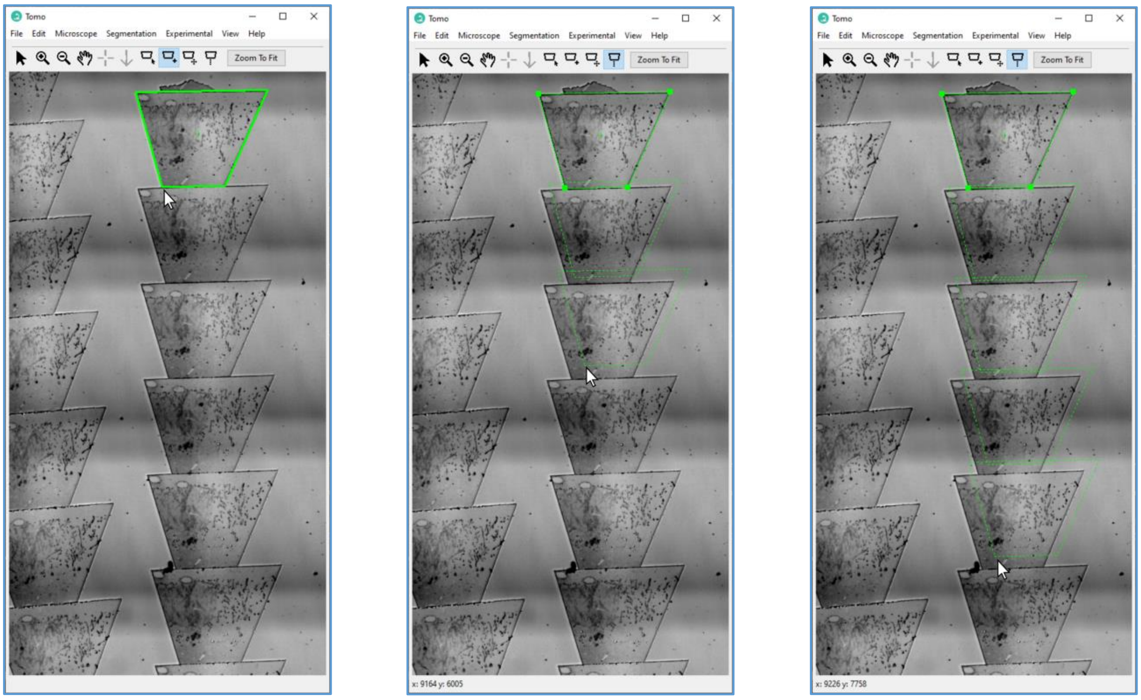

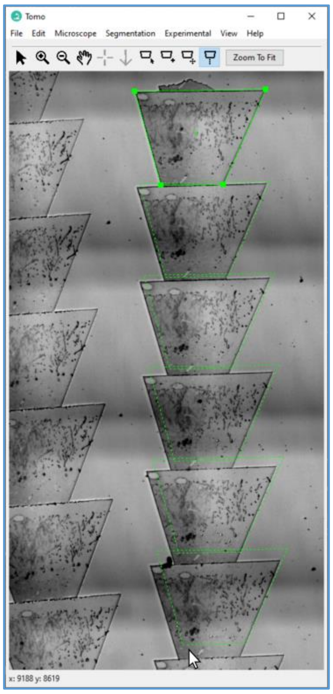

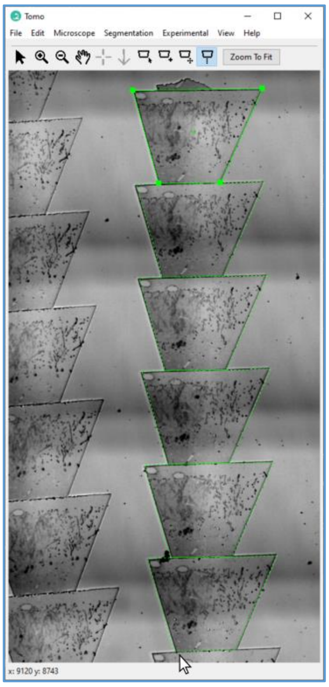

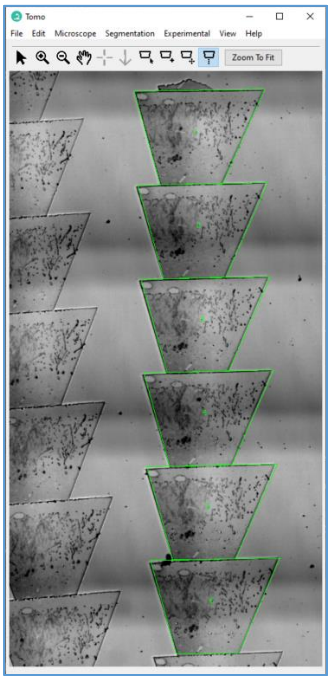

Often, strips of sections created using the Ribbon Builder correspond reasonably well with the true section boundaries that are visible in the overview image, but not exactly. In that case they could be modified manually one by one using the Polygon Editing tool. Fortunately, they can also be corrected semi-automatically using an active contours approach.

A similar shortcoming of the ribbon builder tool in the current version of Tomo is that it only creates *translated* copies of the template polygon. Since it ignores rotation between the sections, it is useful for straight strips but fails for curved strips. Curved strips however, can also be created using the active contours technique.

This powerful active contours approach is explained next.

Active contours for outline improvement and ribbon building

Stated in very simple terms and applied to the current context, active contours is an algorithm that can improve the position and shape of a quadrilateral section outline so that it better matches the true edges of the section in the overview image.

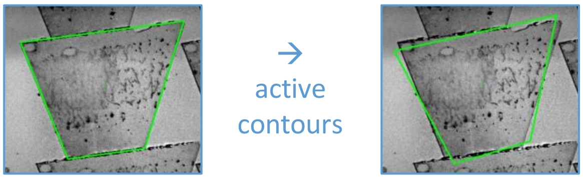

The active contours algorithm can be used to improve a poor manually drawn section outline. It can also be used as an optimization step in any algorithm that produces inaccurate section outline predictions, for example during automatic ribbon building. Tomo uses active contours in both situations. We will now describe how this is done in practice.

In the Tomo menu bar, choose “Segmentation > Find slice contours…” to open the side bar with the active contours tools.

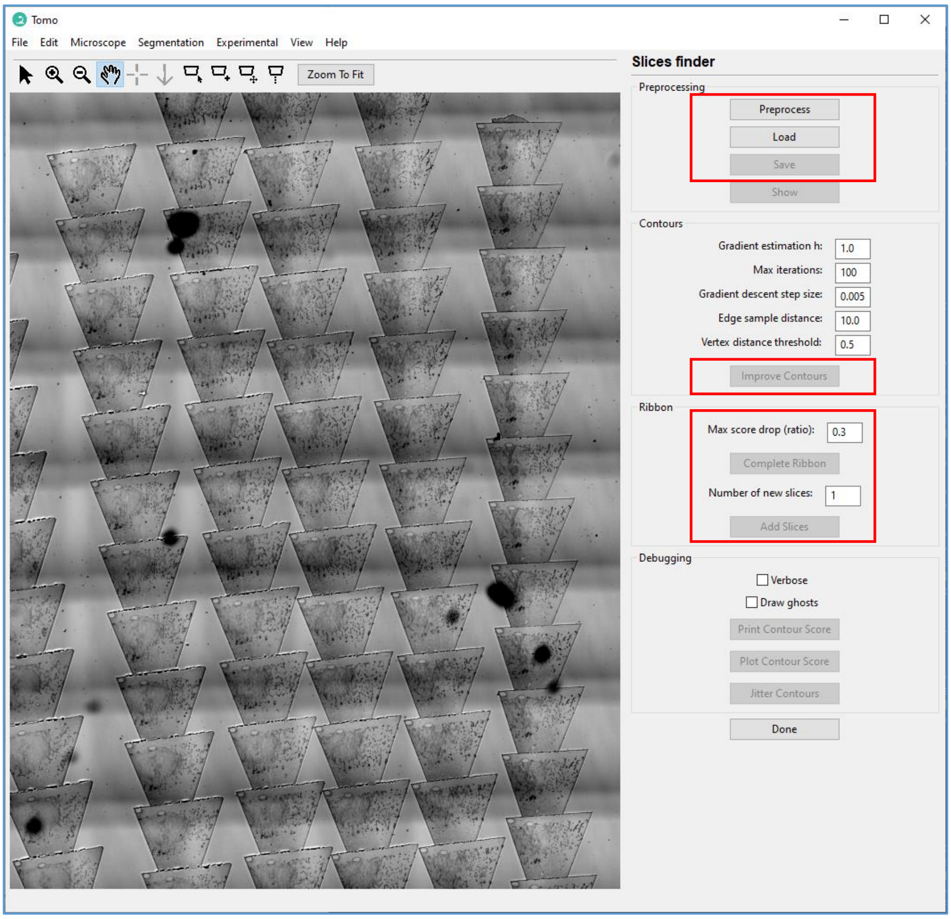

The side bar looks a bit overwhelming, because it offers several parameters that should not actually be modified[5]; hence we will omit their description here. The boxes in red indicate the only part of the user interface that is truly relevant.

Preprocessing

As can be seen from the screenshot above, initially the *Improve Contours*, *Complete Ribbon* and *Add Slices* buttons are all disabled. This is because the active contours algorithm does not actually operate on the raw overview image, but requires a "pre-processed" version of it. This pre-processed overview image can either be loaded (via the *Load* button) if we computed and saved it before in Tomo (using the *Save* button). To calculate it now, click the *Preprocess* button to open an interactive dialog for visually inspecting the effect of the different preprocessing parameters.

[5] They are remnants from the prototype stage of this tool and will be removed in the next version of Tomo.

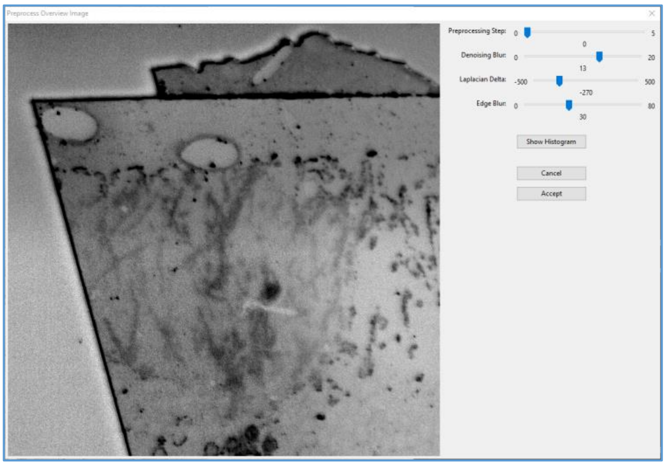

The left side of the dialog shows a close-up from the complete overview image. By clicking and dragging on this preview, the close-up view can be moved around in the overview image. Place the close-up over the edge of a typical section.

We will omit the full technical details here, but the basic goal of preprocessing is to make the active contours tools more robust to noise, and to allow them to better attract section outlines that are initially quite far from their true position. To accomplish this, the raw overview image is first contrast enhanced, then blurred (to remove noise), then edges are enhanced (to improve the accuracy with which the section outlines are recognized), and the image is then blurred once more (to extend the range over which approximate outlines can still be captured by their true edge).

The *Preprocessing Step* slider lets us examine the preprocessed result after each of these steps. In the left most position of the slider (position 0), the raw overview image is shown. Intermediate positions (1-4) show to the cumulative effect of the different preprocessing steps. Typically we are however only interested in the combined visual result of all these steps, and will move this slider completely to the right (position 5).

We can then interactively tweak the *Denoising Blur*, *Laplacian Delta* and *Edge Blur* sliders to obtain a preprocessed result with (ideally) little noise and debris visible, but with clear white-on-black uninterrupted wide section edges.

The images below show the cumulative effect of the preprocessing operations, as well as what a nice preprocessed result looks like.

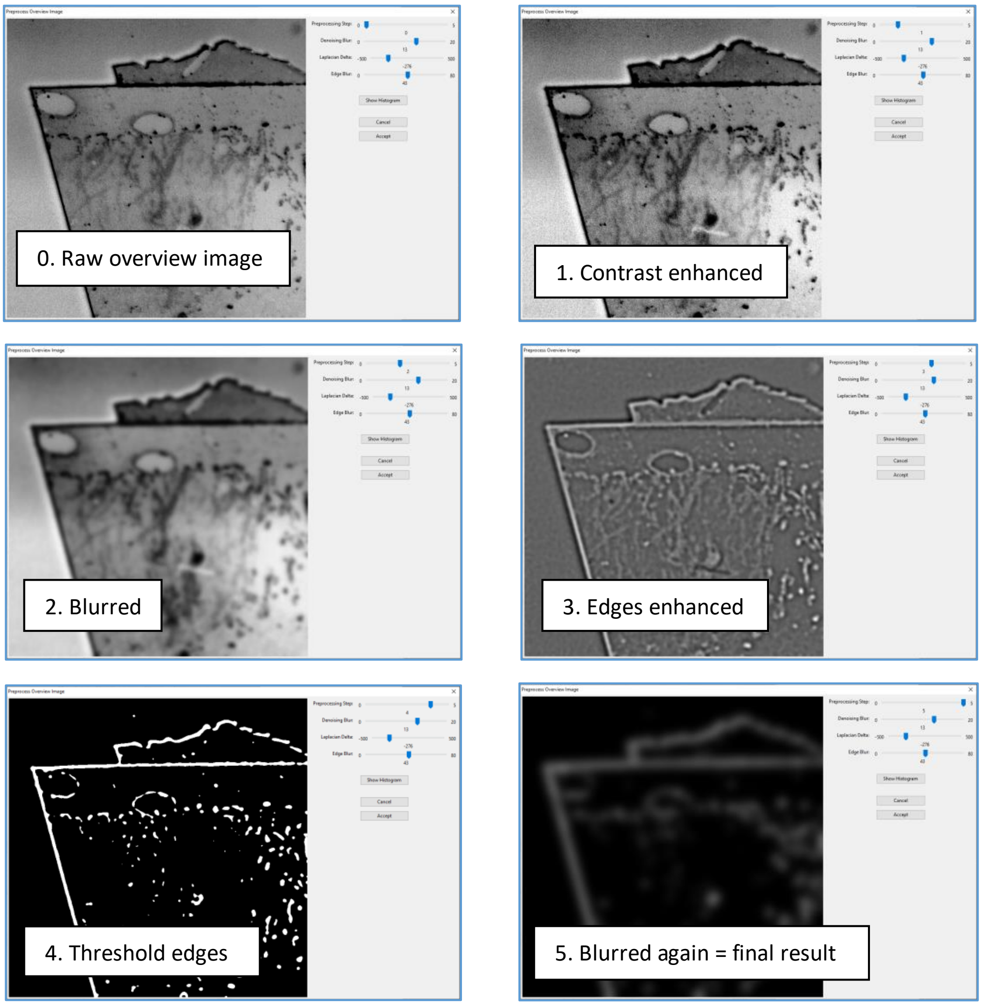

When satisfied with the preprocessed result, press Accept to compute a preprocessed version of the complete overview image.

Improve contours

As soon as the overview image is preprocessed, we can use the automatic contour improvement tool. For example, the corners of the very inaccurate section outline on the left are automatically snapped to the true section boundary simply by pressing the *Improve Contours* button.

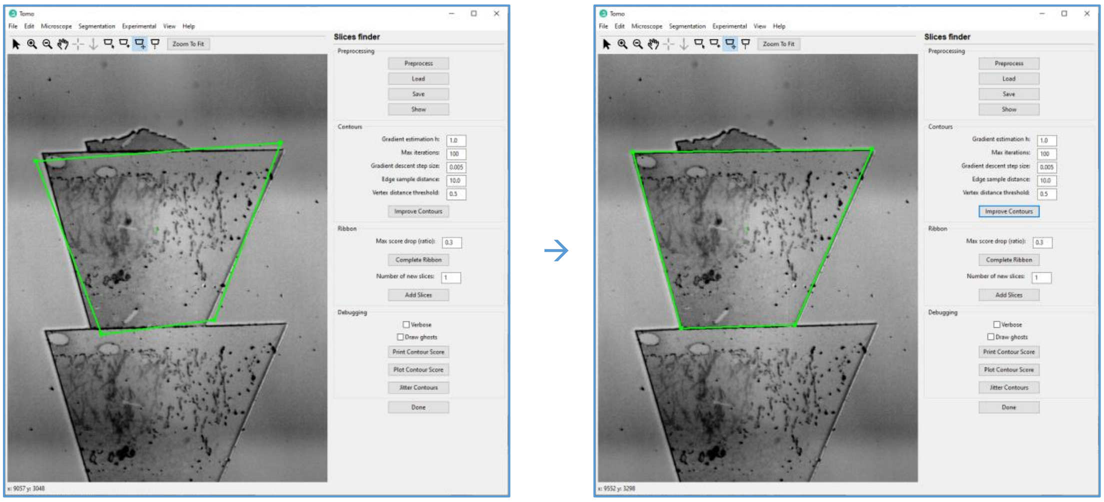

It is also possible to improve several contours at once by selecting them all before pressing *Improve Contours*.

Grow a ribbon

Another interesting use case is the creation of ribbons. We start by selecting a single section outline. We can then either fill in the desired number of additional sections to attach to it and press *Add Slices*. This will iteratively add a section and improve its position using the active contours.

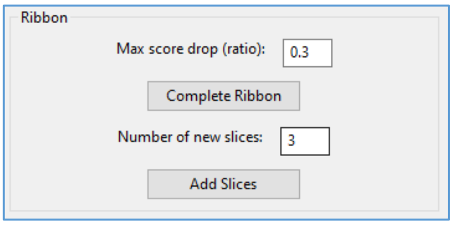

Alternatively, we can just select a single existing section and press *Complete Ribbon*. This will repeatedly add a new section to the previous one, effectively growing a ribbon, until we reach the end of the ribbon.

This concludes are explanation of creating, finding and editing the outlines of all relevant sections on the overview image. When this task is completed, we typically save the outlines to file (File > Save Slice Polygons…). Then we move on to the next step in our pipeline: establishing the precise relationship between stage movement and the overview image in Tomo. We discuss this next.

Aligning microscope stage and overview image

When we loaded the overview image in Tomo we also specified the size of the pixels (in nanometer). This established the correct *scale* between distances in pixels on the overview image, and physical distances of stage movement. What is still not specified, however, is the *translation* between the origin of the overview image coordinate system, and the microscope stage origin. Put differently: where in pixel coordinates on the overview image is the microscope looking at, if we move the microscope stage to its physical origin, stage coordinates (0, 0)? Odemis and Tomo have to be used in parallel to specify this critical correspondence. We will now explain how to do so.

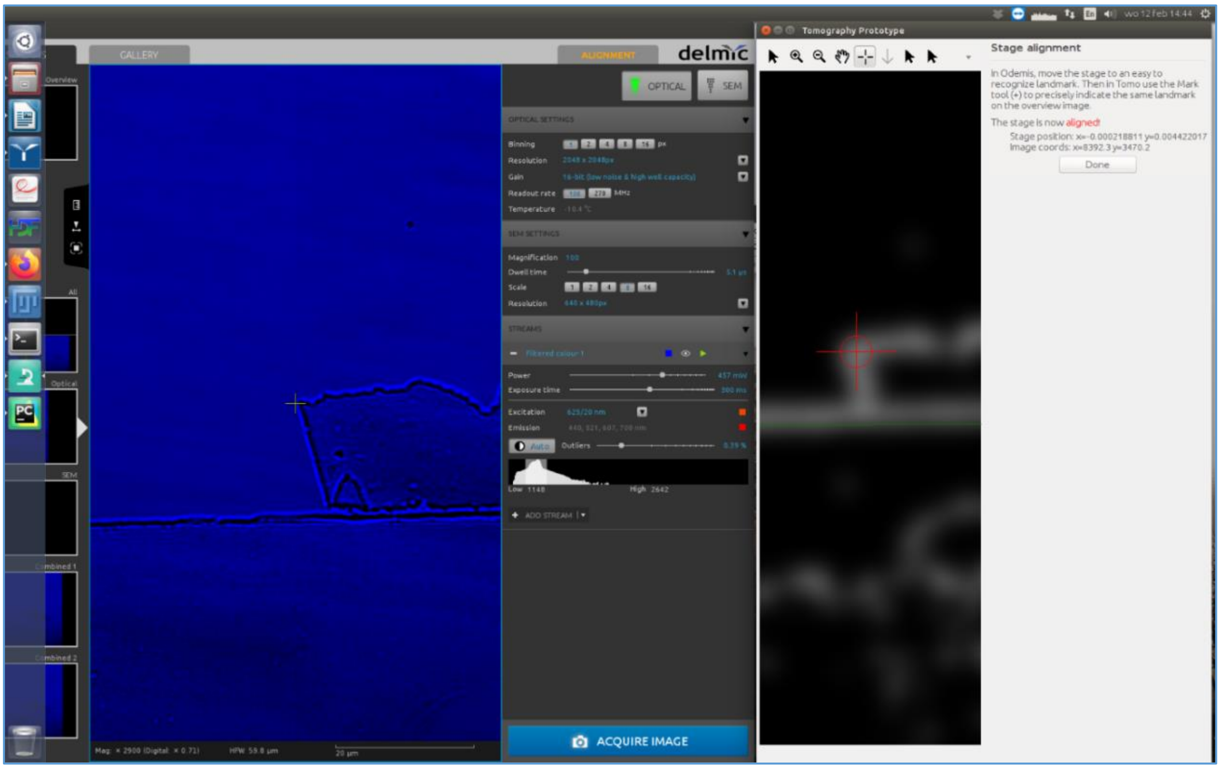

First, in Odemis manually position the LM microscope precisely over an easy to recognize sample landmark.

Then, in Tomo, select “Microscope > Align stage and overview image…” to pop up the Stage Alignment sidebar. Use the zoom and pan tools in Tomo to locate the exact same sample landmark on the overview image, and with the Mark tool ( ) click on landmark to mark it. A red crosshair will be drawn over the landmark as feedback to the user. Finally, press Done to dismiss the side bar.

Tomo now has all the necessary information to convert between absolute physical stage positions and pixels on the overview image.

Building a focus map

Accurate focus control is obviously important for microscopy. In Tomo we aim to acquire images on many sample sections, but the sections may vary slightly in thickness, and the holder for the sections may not be perfectly horizontal. To make sure each image is in focus, we can resort to auto-focus.

Alternatively, for samples with little contrast where auto-focus might fail, we implemented a focus-map in Tomo. It may result in sharper images acquired in transmitted light conditions, which also makes automatic alignment of the acquired images more robust.

Using the focus map is optional. It can only be used for LM, not for EM image acquisition.

To build a focus map, select “Microscope > Build focus map…” from the menu in Tomo. The Focus Map side panel will appear.

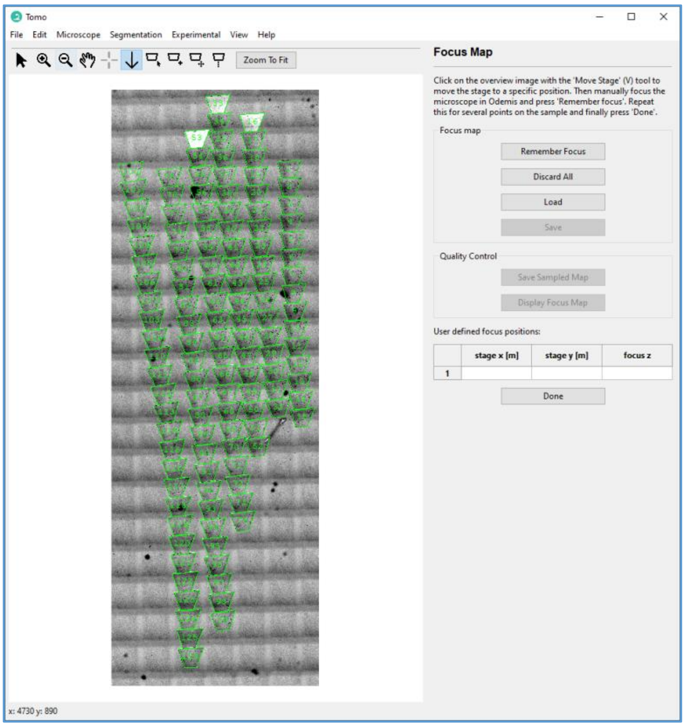

The side bar shows a tabular representation of the focus map: the (x, y) stage position expressed in meters, and corresponding focus z value for each point in the map. The user does not need to enter any numerical values for x, y or z in the table but will simply click on the overview image, and Tomo will query the corresponding x, y and z position values from Odemis, as explained below.

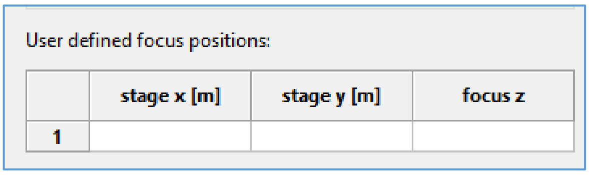

Initially, the focus map table is empty. To add points to the focus map, we will use Tomo in combination with Odemis as follows. In Tomo use the Move Stage tool

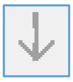

to click somewhere on the overview image where we want to manually acquire focus. Tomo will then calculate the corresponding physical stage position and instruct the microscope to move that spot exactly under the objective. Then, in the Odemis application, we manually adjust the focus so that spot is imaged as sharp as possible. (Tip: to make *small* focus adjustments in Odemis, hold down the shift key while pressing the right mouse button. Note that due to the immersion oil, it takes a little while for the image to stabilize after focus changes.) Then in Tomo, click the Remember Focus button in the Focus Map side panel to add this physical position and its focus position to the focus map. The focus map table will be updated.

This process is repeated for several points across the overview image. Focus points in the focus map are numbered, and indicated in red on the overview image.

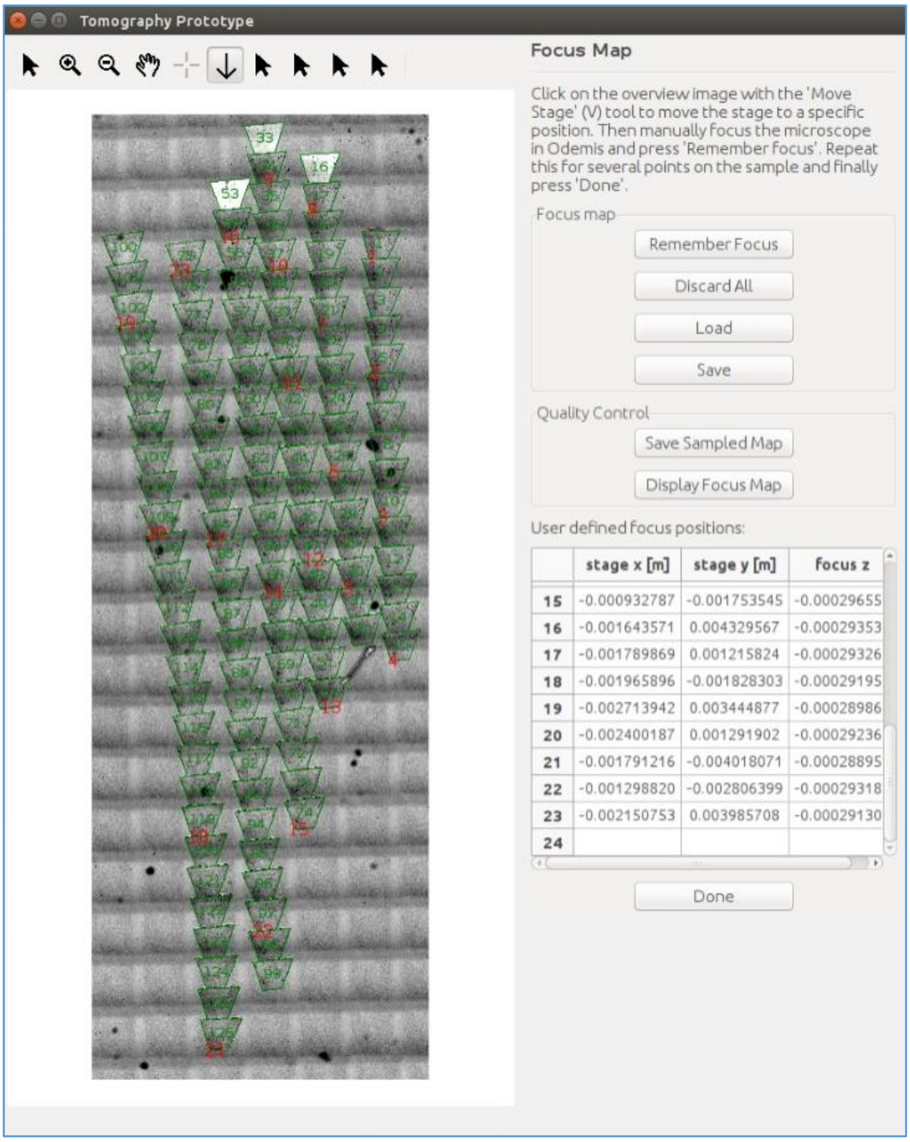

Focus points can be deleted via a right mouse click on the corresponding row in the focus map table. To start over completely, the focus map can be cleared by pressing the Discard All button.

A focus map can also be saved (to a simple text file) and loaded afterwards via the Load and Save buttons.

A graphical representation of the interpolated focus map grid can be shown with the Display Focus Map button. The raw focus values for this grid can be saved to a text file with Save Sampled Map.

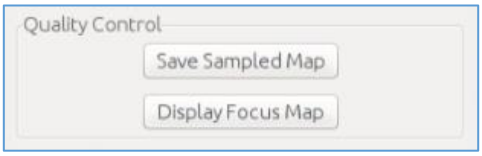

Specifying the region-of-interest

The goal of Tomo is automated imaging and tracking of a user-specified region-of-interest (ROI) across a large number of successive sample sections. After the previous steps we can now finally specify our desired ROI in Tomo.

Open the side panel dedicated for this purpose via “Microscope > Set point of interest…” Then use the zoom and pan tools of Tomo’s canvas to locate and zoom in on the desired sample feature that we want to image. This feature must be identified on the *first* section where we want imaging to start. Once this feature is visible in close up on the overview image, use the Mark tool ( ) to mark this position as our region-of-interest.

Tomo will then automatically predict the approximate corresponding region of interest in the successive sections. These regions-of-interest will be indicated with a red cross on the overview image. Only one region-of-interest (typically spanning multiple sample slices) can be specified. To change the current region-of-interest, simply specify it again with the Mark tool.

The user can also specify the number of successive slices on which this region-of-interest will be tracked and imaged later on. By default it will be tracked across all slices.

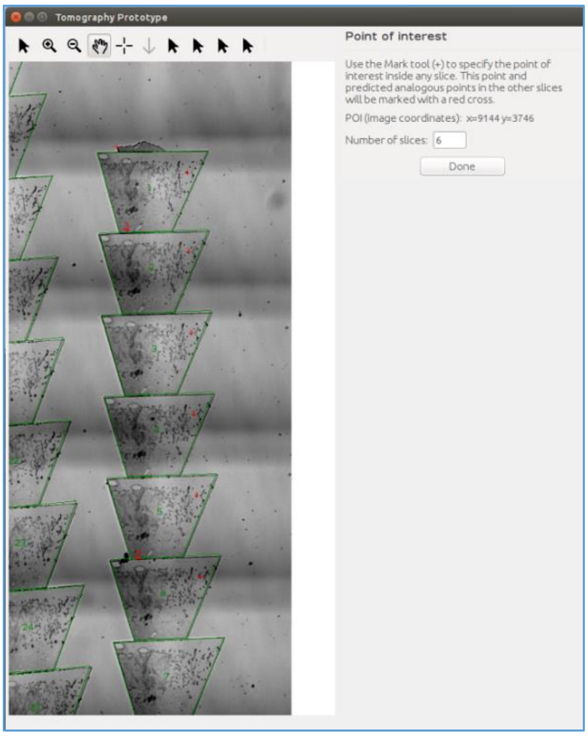

Light microscope image acquisition

We can now start semi-automated acquisition of LM images at the predicted region-of-interest on a series of successive sections. Open the acquisition dialog via “Microscope > Acquire LM Images…”

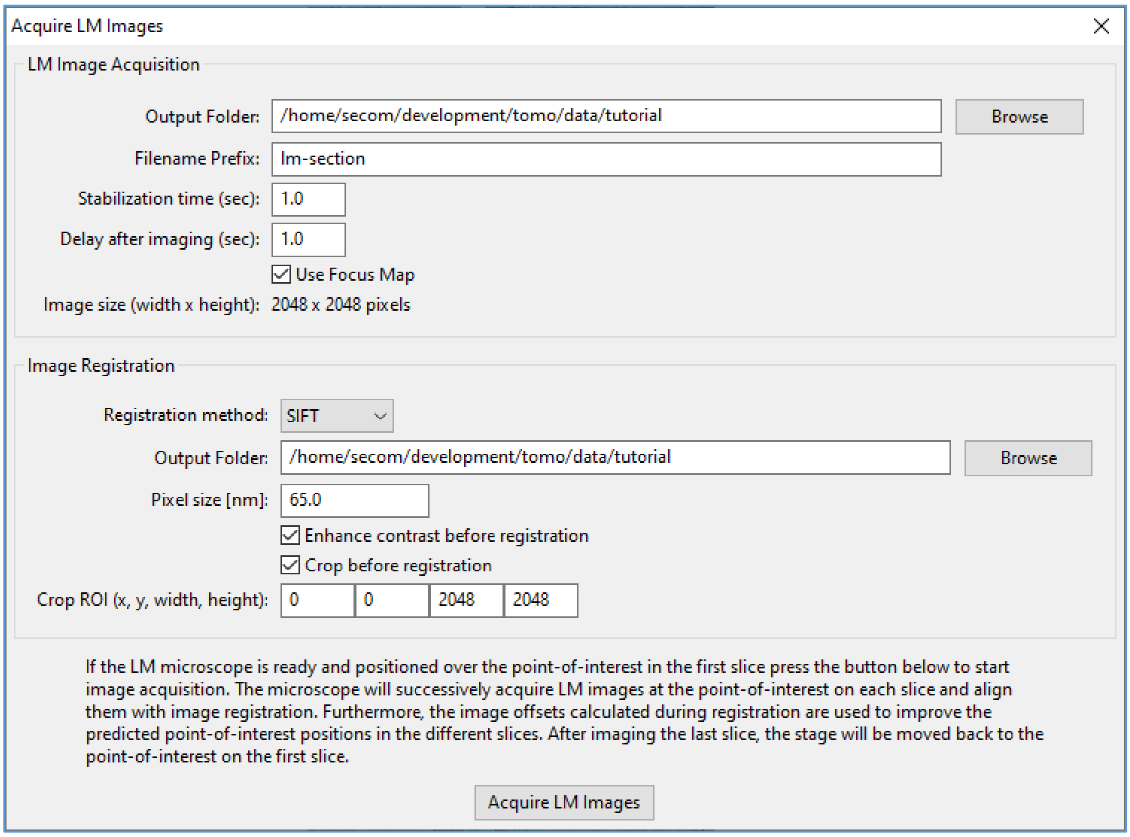

This dialog looks a bit overwhelming, but can be thought of as two halves. The top half lets us specify parameters for **LM image acquisition**, the bottom half parameters for **image registration** of the resulting acquired images. This corresponds to the two phases in our proposed semi-automatic image acquisition pipeline: first we acquire images at the predicted region-of-interest position in each of our successive sample sections (one image of the ROI per section), and next we will perform image alignment (“registration”) of these images. From the observed amount of *mis*alignment Tomo can infer correction distances that will let us localize the ROI in each section with higher accuracy during a next imaging round, either at the same or at a higher magnification. This ROI position correction is especially important if the next imaging round is done with a significantly higher magnification, for example when switching from LM to EM imaging of the same ROI.

We now have a closer look at the different parameters in the dialog.

LM Image Acquisition

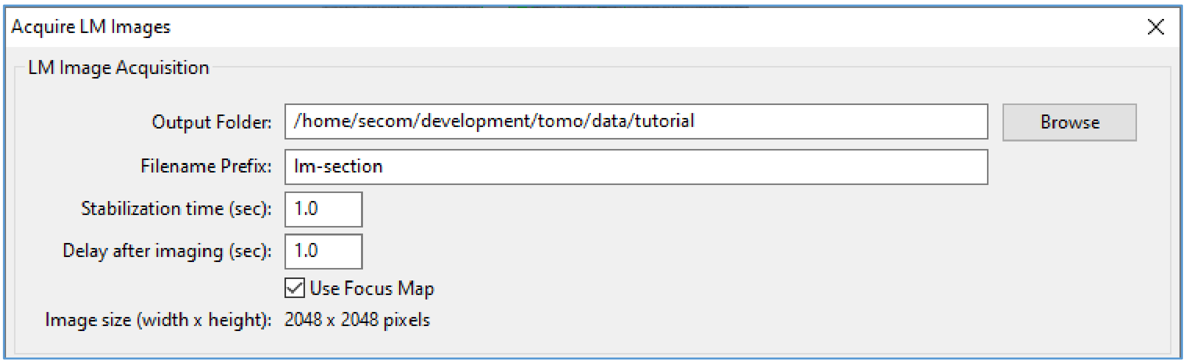

The *Output Folder* is the directory where the raw acquired LM images will be stored. The filename of the images will be the specified *Filename Prefix* followed by the number of the section.

The *Stabilization time* is the time that Tomo will wait after it has moved the stage and (possibly) changed the focus, before actually acquiring the image. A small stabilization delay is advised especially when using an 100x oil immersion lens, to allow the viscous oil droplet between sample and objective to settle.

After requesting the LM microscope to acquire the image, we can additionally ask Tomo to pause for a short time after imaging. This *Delay after imaging* was introduced in Tomo as a precaution to avoid flooding Odemis with imaging requests, but is most likely redundant[6].

If the user built a Focus Map, we can ask Tomo to use it during image acquisition via the *Use Focus Map* checkbox. A focus z-value will then be interpolated from the focus map at the position of the ROI for each image, and set on the LM microscope before acquiring each image. If no focus map is used, all LM images will be acquired at the focus z that the LM microscope was set to before image acquisition is started.

Finally, the *Image size* field is purely informative. It indicates the fixed image size of the images that are being acquired.

Image Registration

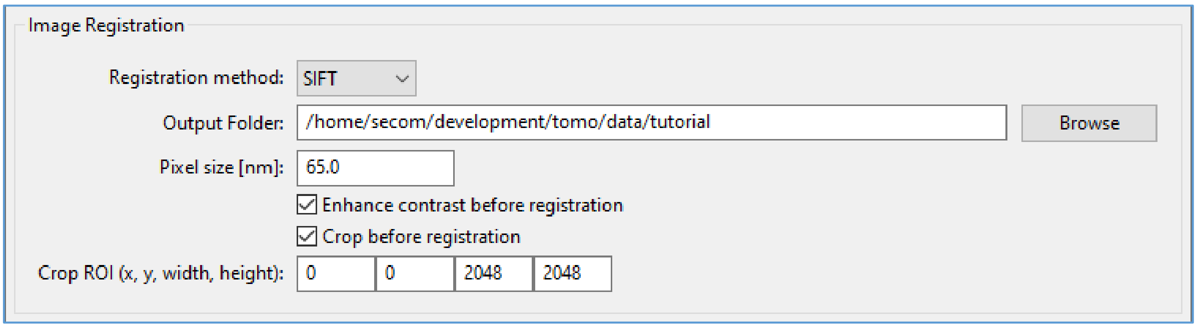

In this section of the dialog we can specify which image *Registration method* to use to align the LM images of the successive slices. Alignment is done in the background after all images have been acquired. Tomo will then start Fiji in the background and run a Fiji plugin to do the alignment.

---

[6] This optional delay will be removed from the next version of Tomo.

Currently two well-known image registration plugins for Fiji are supported: "Linear stack alignment with SIFT" and "StackReg". The former was found to work well for LM images. Alignment is restricted to rigid transformations only.

The registered images will be saved to a single TIFF file with multiple z-planes, one z-plane per section. This TIFF file will be called "SIFT_aligned_stack.tif" or "StackReg_aligned_stack.tif" and saved to the user-specified *Output Folder*. Additionally, the unaligned stack will be saved there too, as "unaligned_stack.tif".

If the *Enhance contrast before registration* checkbox is checked, the raw images will be contrast enhanced before applying image registration. We found that this increases the reliability of the image alignment.

Finally, if the sample has a lot of "debris" that is unrelated to our ROI, this may confuse the image registration algorithms, resulting in incorrectly registered images. As a workaround in this situation, the user can optionally perform a crop on the acquired images, keeping only the actual ROI and discarding the debris. This is requested via the checkbox *Crop before registration*. By default this checkbox should be off.

If cropping is requested, a *Crop ROI* rectangle must be specified as the coordinates of its top-left corner within the image, and its width and height. The top-left corner of the original image is (0,0) with the y-axis pointing downward. As a (somewhat contrived) example, suppose we desire to crop 10% along the 4 edges of the acquired images. Then we would need to specify a crop rectangle with (X,Y)=(205, 205) and (width, height)=(1638, 1638) on the raw 2048 x 2048 pixel images, as shown on the figure below.

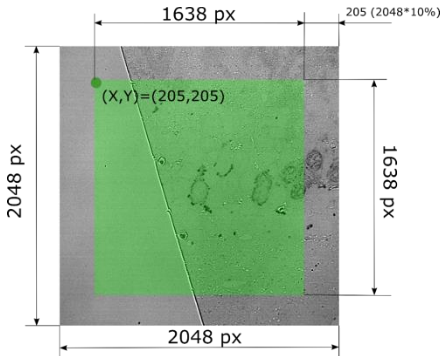

Performing the LM image acquisition

The parameters described above modify aspects of the semi-automated tomography pipeline that Tomo controls. The low-level microscope settings must however be specified in Odemis. So the user must now set the LM microscope acquisition parameters in Odemis, such as:

- Exposure time
- Laser power
- Histogram

Furthermore, <u>it is absolutely mandatory that the laser is turned on in Odemis in the Optical Tab,</u> otherwise the acquisition will record dark images.

We are now ready to start acquiring images, so press the “Acquire LM Images” button!

Verifying the resulting registered image stack

When LM acquisition and stack alignment have completed, Tomo will pop up a dialog asking to verify if registration was successful.

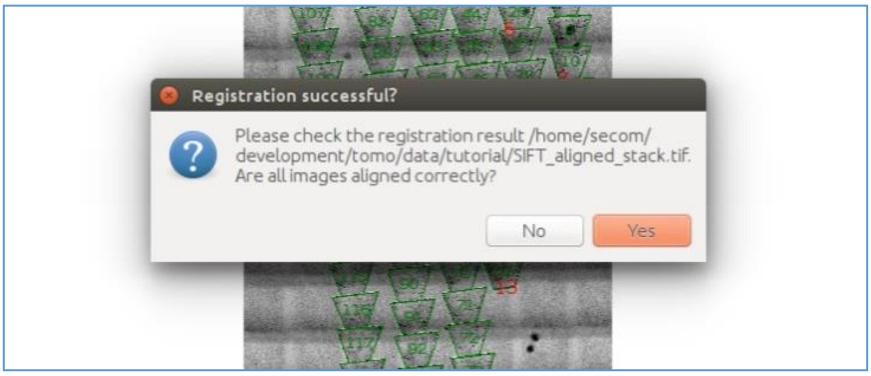

The user can now open the aligned image stack (for example in Fiji) and visually verify if automatic image alignment was performed correctly. If registration was indeed done correctly, we can press Yes to dismiss the confirmation dialog, which then concludes LM image acquisition.

If however, image registration failed, we press No, and a new dialog will appear asking the user to perform the registration manually in Fiji with the “StackReg” or “Linear Stack alignment with SIFT” plugins. Manual stack alignment can be more powerful because we then have access to the full range of parameters to tweak the registration algorithm. After manual registration in Fiji, save the log output generated by the registration plugin to a text file, and specify this file in the open registration dialog in Tomo. Tomo will then parse this log file and extract the image registration transformations required to improve its ROI position correction.

Electron microscope image acquisition

After acquiring at least one LM image stack (which also is used to improve our region-of-interest position prediction), we can move to even higher magnification and resolution by switching to EM image acquisition.

Before we start however:

<u>It is absolutely mandatory that the SEM data stream is turned off in Odemis otherwise the EM image acquisition will yield bogus images containing only noise. To turn off the SEM stream, go to Odemis, and press the SEM button there.</u>

Then in Tomo, open the EM image acquisition dialog via “Microscope > Acquire EM Images…” A dialog appears which is quite similar to the one for LM image acquisition which we discussed before.

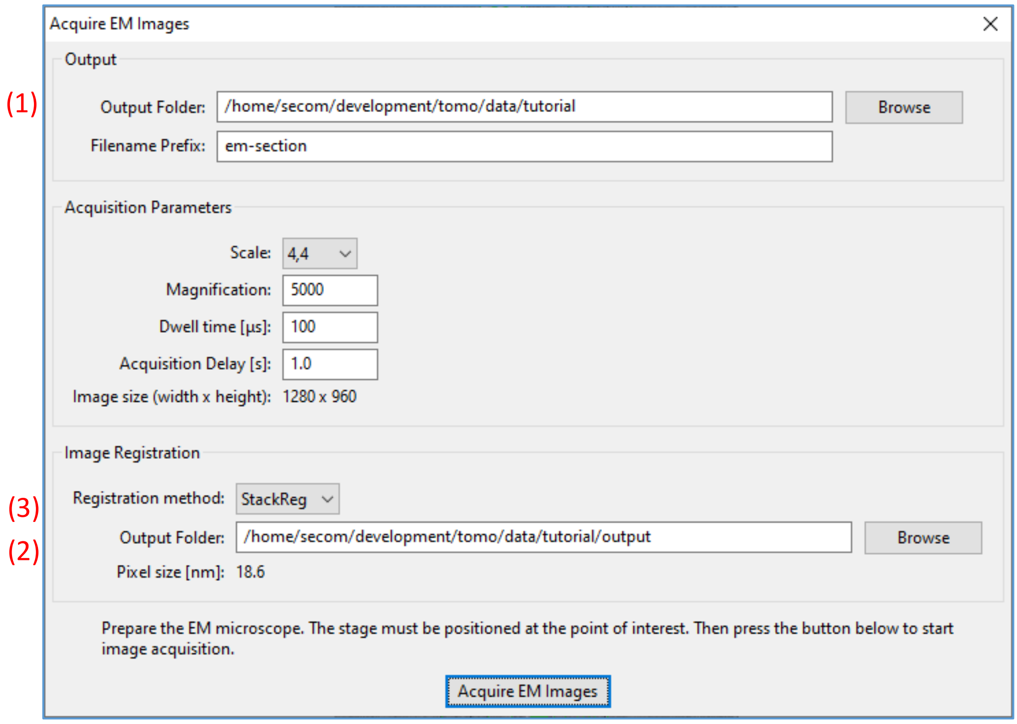

Again we can specify an *Output Folder* (1) for the individual acquired EM images, and an *Output Folder* (2) where the image stack will be saved after registration.

We can also specify the algorithm (3) that will be used for aligning the EM images after acquisition. Both SIFT and StackReg are supported, but we found StackReg to work best for EM images. Alignment is restricted to rigid transformations only.

We must specify the dwell time, magnification and scale of the image (suggested value 4,4) and acquisition delay in the *Acquisition Parameters* section of the dialog.

The low-level EM microscope parameters, however, can only be set outside Tomo. The user must set the parameters of the SEM (acceleration voltage, probe current, type of detector, astigmatism, Focus,…) in the JEOL PC-SEM Windows application running on a separate computer.

Finally, we can now press the "Acquire EM images" button to start EM image acquisition.

After automatic EM image acquisition and registration, the user will be asked to visually inspect the resulting aligned EM image stack. If automatic registration of the EM images was not successful, the user can proceed in the same fashion as explained in the section on LM image acquisition above to perform a manually image alignment.
