# Supplementary figures and images for: A workflow for streamlined acquisition and correlation of serial regions of interest in array tomography
Source: BMC Biol. 2021 Jul 30;19:152. doi: 10.1186/s12915-021-01072-7 (PMC8323292; doi:10.1186/s12915-021-01072-7)

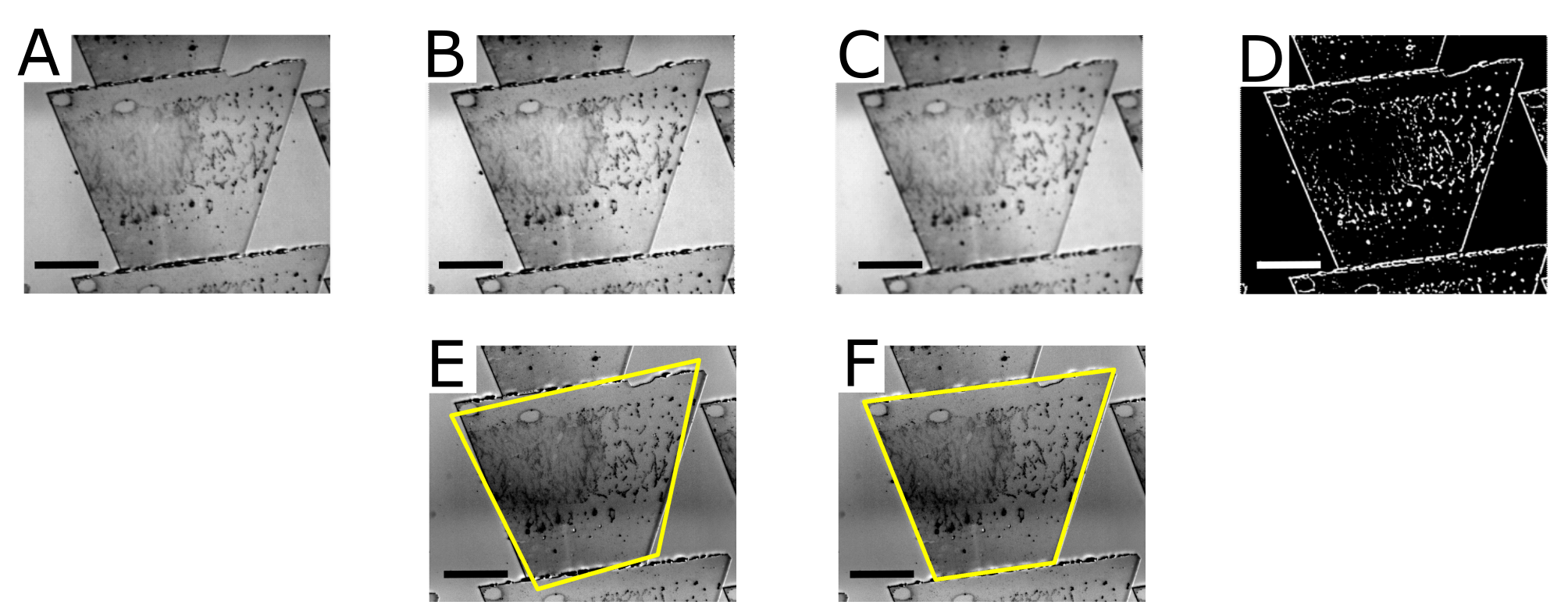

Supplement: Supplementary file 3 — Additional file 3: Figure S1. Image processing steps used to detect sections. From left-to-right, the subsequent steps in the processing pipeline are shown. a Close-up of the unprocessed light microscope overview image. b Image after contrast normalization. c Noise reduction via Gaussian blurring and d a Laplacian filter are applied. e Approximate outline of a sample section roughly sketched by the user. f Outline after active contours optimization. The resulting contour matches the true section outline nicely. Scale bar is 100 μm. [file 12915_2021_1072_MOESM3_ESM.png]

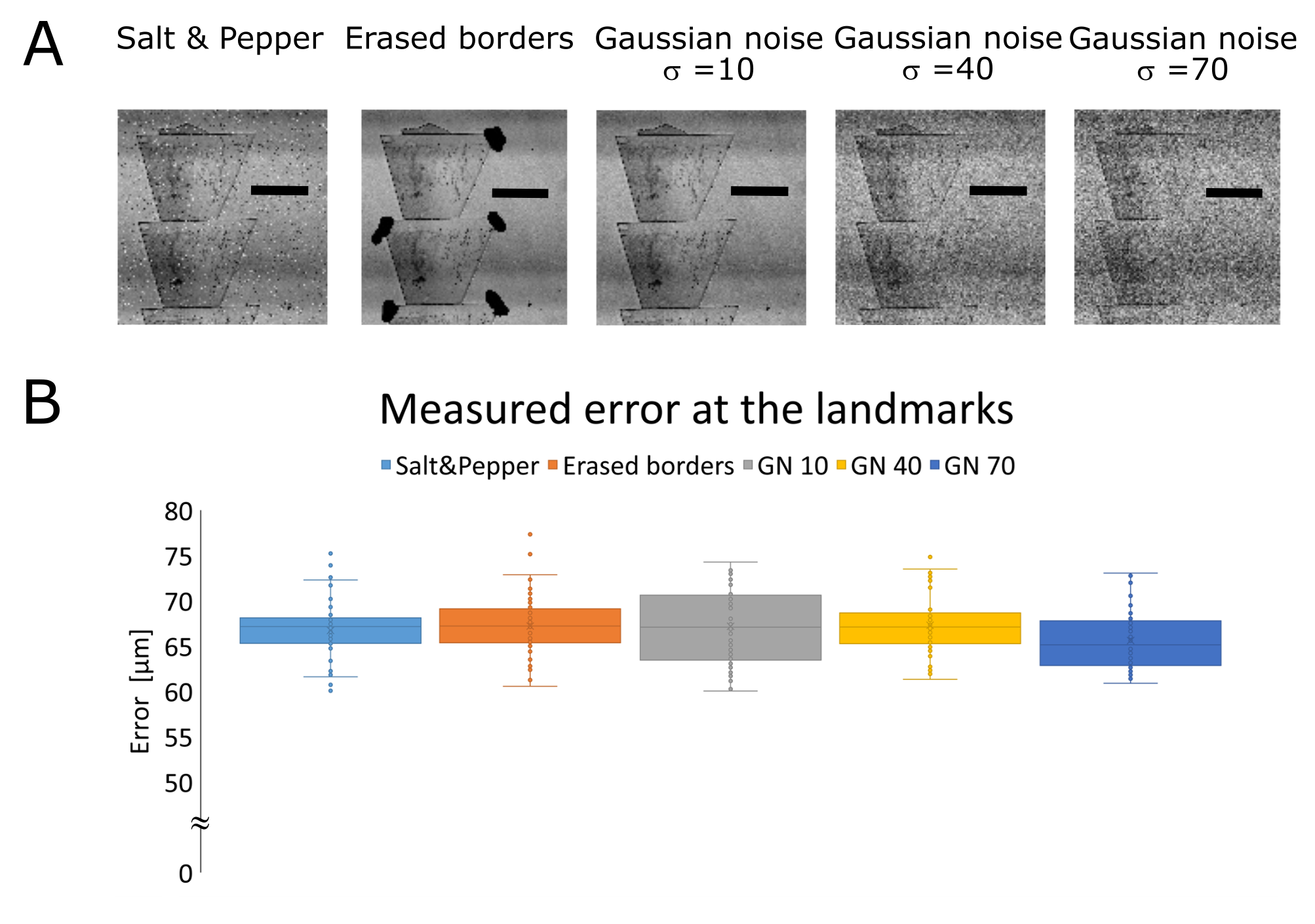

Supplement: Supplementary file 4 — Additional file 4: Figure S2. Section detection tested with intentionally degraded images. a Modified images. From left to right: ground truth original overview image, added salt and pepper noise, erased corners and borders, (GN 10) added Gaussian noise σ=10, (GN 40) added Gaussian noise σ=40, (GN 70). b Whisker box plot of the tested images with added noise and removed corners and borders. Errors of the boarders of the sections of the first ribbon (see Fig. 3) are shown. Scale bar 200 μm. [file 12915_2021_1072_MOESM4_ESM.png]

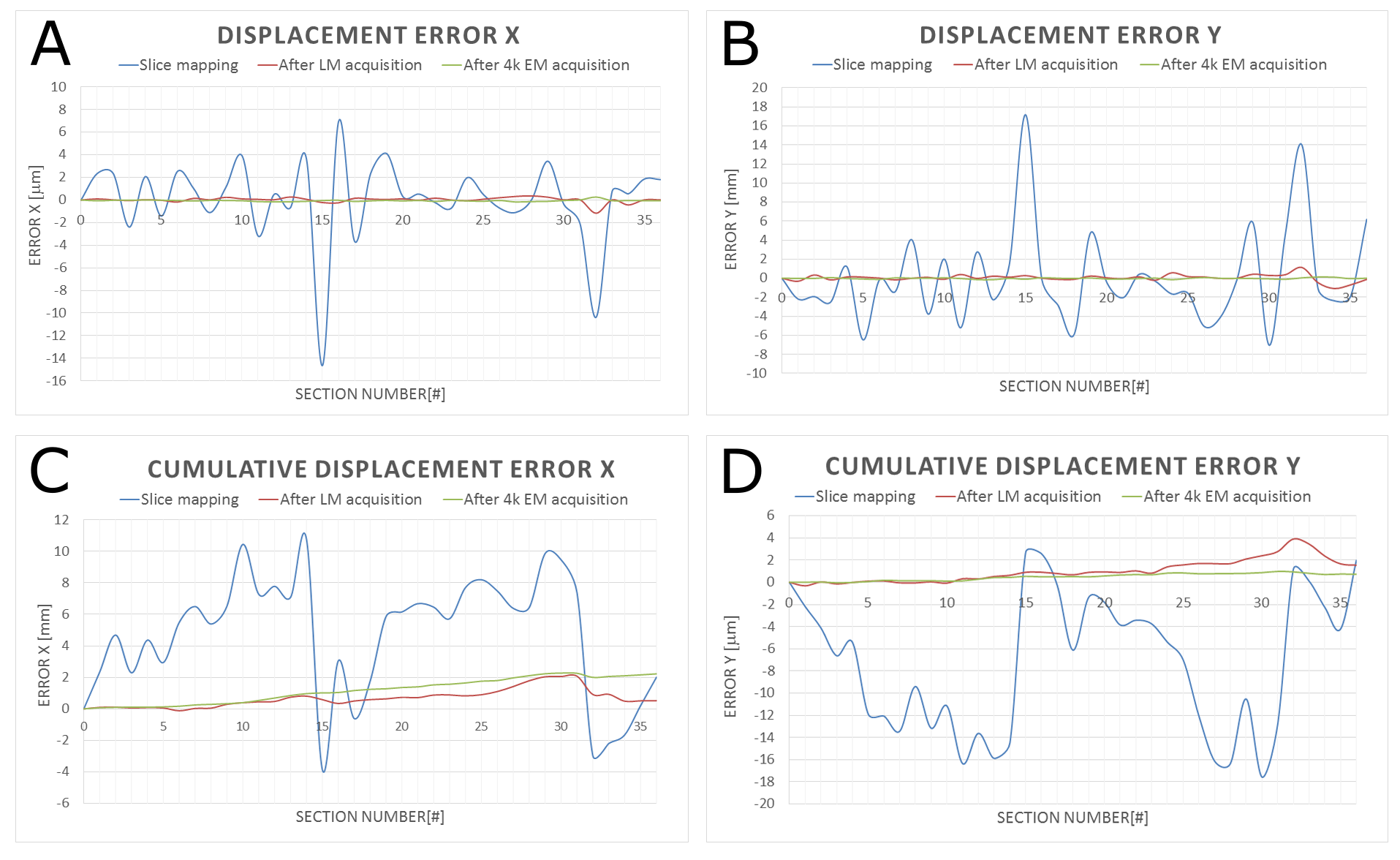

Supplement: Supplementary file 7 — Additional file 7: Figure S3. Comparison of displacement errors with increasing magnification. a Displacement error in x [μm] and b in y [μm] at each section for slice mapping on the 20x overview image and after LM acquisition with 100x lens and 4kx EM acquisition, compared to corrected navigation after 12kx EM acquisition. c and d showing the respective accumulated error of a and b. Representative data is shown. [file 12915_2021_1072_MOESM7_ESM.png]

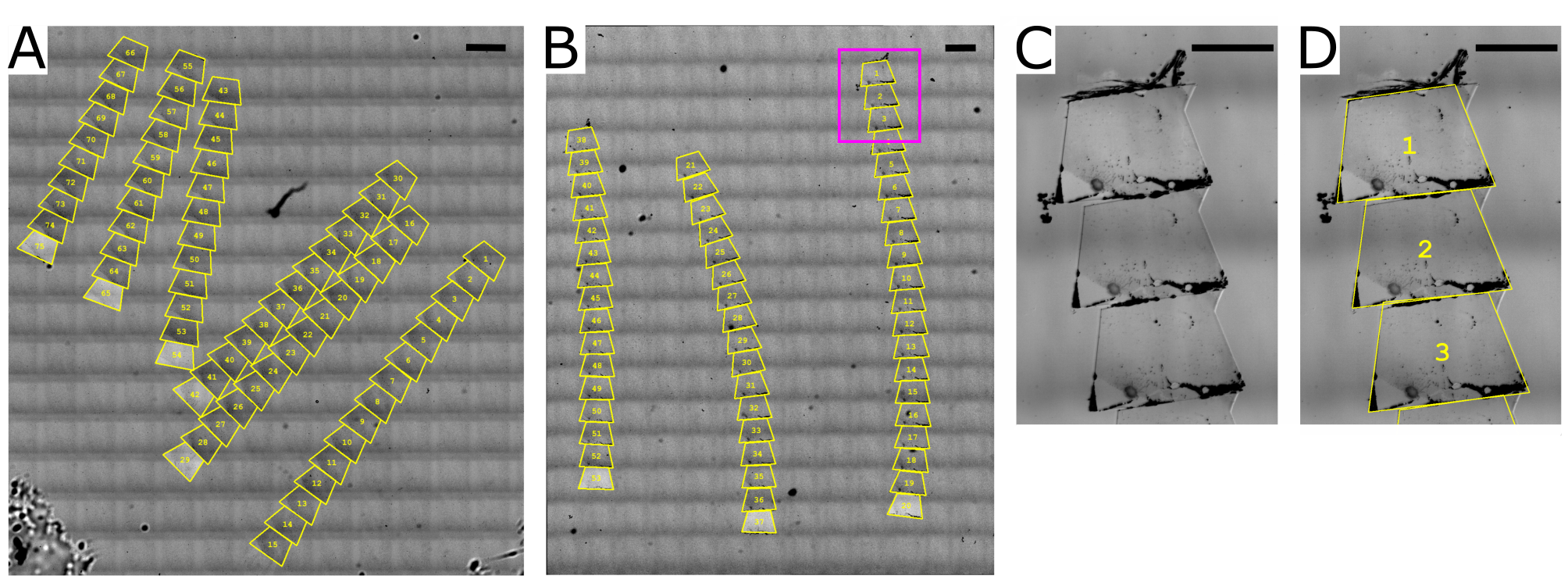

Supplement: Supplementary file 8 — Additional file 8: Figure S4. Effect of poor sample preparation on Tomo performance. a Ribbons with a good quality boundary but inclined distribution on the ITO coverslip. b Curved ribbons with multiple defects. c Detail from b, indicating glue contamination in black and additional wedges on the top right side of the sections causing deviation from the desired trapezoidal /quadrilateral shape. Scale bar a, b= 500 μm, c 250 μm. [file 12915_2021_1072_MOESM8_ESM.png]
