# Supplementary material for: A workflow for streamlined acquisition and correlation of serial regions of interest in array tomography
Source: BMC Biol. 2021 Jul 30;19:152. doi: 10.1186/s12915-021-01072-7 (PMC8323292; doi:10.1186/s12915-021-01072-7)
Supplement: Supplementary file 10 — Additional file 10: Table S1. Comparison of Tomo features with other state of the art software solutions. Tomo, MAPS, Atlas, Mosaic Planner, and Wafer Mapper are compared. [file 12915_2021_1072_MOESM10_ESM.docx]

|  | Tomo | MAPS | Atlas | Mosaic Planner  [20] | Wafer Mapper [9] |
| --- | --- | --- | --- | --- | --- |
| correlation/ overlays | inherent in iLEM | through stage information and image based | through stage information and image based | through TrakEM2 plugin from Fiji | no LM, no correlation |
| stitching | yes | no | yes | yes | yes |
| section detection | active contours on geometric shape | automatic template matching | manual clone tool and snapping by template matching | no | image treatment of Wafer overview and user thresholding for centroid section detection |
| navigation for ROIs | coordinates used throughout incl. LM used (fast) | SEM stage and SEM overview | SEM stage and SEM overview | coordinates from LM co-localization | coordinates from aligned stack of section overview images. Mosaic acquisition |
| correction for distortions/ shape change of sections | finite element approach | - | - | - | - |
| ROI tracking/ alignment | iterative / stepwise refinement | based on raw section shape, one-time offset correction (called refinement) | based on raw section shape | based on linear projection optimized by co-localization | based on section overview stack alignment and mosaic at ROI stack alignment |
| section shape | quadrilateral | any /? | any / ? | any | any |
| result | pyramidal volumes from low to high resolution | overview + high res. stack | overview + high res. stack | stack of LM images used to be aligned with low mag EM | Wafer overview, section overview stack, high res. EM mosaic at target point |

**Table S1. Comparison of Tomo features with other state-of-the-art software solutions.**
